# Continued fractions and the Thomson problem

**DOI:** 10.1038/s41598-023-33744-5

**Published:** 2023-05-04

**Authors:** Pablo Moscato, Mohammad Nazmul Haque, Anna Moscato

**Affiliations:** 1 School of Information and Physical Sciences, The University of Newcastle, Callaghan, NSW 2308 Australia; 2 The Australian National University, Canberra, ACT 2600 Australia

**Keywords:** Physics, Mathematics and computing, Computational science

## Abstract

We introduce new analytical approximations of the minimum electrostatic energy configuration of *n* electrons, *E*(*n*), when they are constrained to be on the surface of a unit sphere. Using 453 putative optimal configurations, we searched for approximations of the form $E(n) = (n^2/2) \, e^{g(n)}$ where *g*(*n*) was obtained via a memetic algorithm that searched for truncated analytic continued fractions finally obtaining one with Mean Squared Error equal to ${5.5549 \times 10^{-8}}$ for the model of the normalized energy ($E_n(n) \equiv e^{g(n)} \equiv 2E(n)/n^2$). Using the Online Encyclopedia of Integer Sequences, we searched over 350,000 sequences and, for small values of *n*, we identified a strong correlation of the highest residual of our best approximations with the sequence of integers *n* defined by the condition that $n^2+12$ is a prime. We also observed an interesting correlation with the behavior of the smallest angle $\alpha (n)$, measured in radians, subtended by the vectors associated with the nearest pair of electrons in the optimal configuration. When using both $\sqrt{n}$ and $\alpha (n)$ as variables a very simple approximation formula for $E_n(n)$ was obtained with MSE= $7.9963 \times 10^{-8}$ and MSE= 73.2349 for *E*(*n*). When expanded as a power series in infinity, we observe that an unknown constant of an expansion as a function of $n^{-1/2}$ of *E*(*n*) first proposed by Glasser and Every in 1992 as $-1.1039$, and later refined by Morris, Deaven and Ho as $-1.104616$ in 1996, may actually be very close to −1.10462553440167 when the assumed optima for $n\le 200$ are used.

## Introduction

Very recently, a number of papers have been related to uncovering equations that model physical systems with high accuracy. The denomination of ‘precision machine learning’ has been suggested for this research field^1^. An increased interest now exists in the research and development of new methods for symbolic regression, a technique for multivariate regression problems that also has a history as a hub between the machine learning, computational intelligence and evolutionary algorithms communities.

While many pioneer works exist in the area, since the publication of the work of Schmidt and Lipson in 2009 in the journal *Science*^2^, it is without question that many researchers have found its outcomes as a source of inspiration in Physics. Their work then led to the development of a commercial software (called *Eureqa*)^3^, and thanks to their free academic license, the power of the genetic programming heuristic running as a search engine, and the easy-to-use graphical user interface, this spawns the interest of many newcomers into the field.

In the original paper by Schmidt and Lipson, the objective of the authors was to show how symbolic regression can be used to uncover natural laws of Physics from experimental data. Far from being a mathematical curiosity in the field of classical mechanics, identifying invariants in the dynamics of systems and uncovering new empirical laws is still pretty much a constant need in science. Like in the case of Kepler, who established three laws for the movement of the celestial bodies, those laws may not stand forever, but they may inspire the work of theoreticians, particularly when the fitting of the observed data by the empirical law is relatively simple and also pretty accurate. In that case, it was Newton who simplified the three laws and later established his simpler theory. Today we have a similar process going on; sometimes, many features are measured from experimental settings, and since symbolic regression methods are generally coupled with model complexity reduction heuristics, a subset of the variables is generally chosen together with a good model. This allows theoreticians to concentrate perhaps on those variables in the quest to find a simpler explanation to the phenomenon of interest.

More recently, an MIT group has published another machine-assisted method to uncover relationships in Physics^4^. The approach is properly called *AI-Feynman* because its claim is to have been able to “uncover” 100 equations from the three-book series called *The Feynman Lectures on Physics* by Feynman, Leighton, and Sands as well 90 percent of another set of 20 “more challenging” equations coming from other Physics collections. In a new preprint, the new version called *AI-Feynman 2.0* is shown to be superior to the original approach^5^ including robustness to experimental noise.

One of the fields that well illustrates on the benefits from symbolic regression is Astronomy, an area of science with a long tradition in data-driven discovery. In 2014, Krone-Martins et al. reported “the first analytical expression to estimate photometric redshifts” suggested by a computer algorithm^6^, and a year before a group at Caltech illustrated on the use of symbolic regression to uncover classical astronomical relationships like the Hertzsprung-Russell diagram, the fundamental plane of elliptical galaxies, and the period-amplitude plane of RR Lyrae stars^7^. These two groups have also used the academic version of Eureqa, and for many years, the company website collected information on several new applications of symbolic regression, including our own using their software, in areas as diverse as consumer behavior and business analytics^8–10^ and drug response in cancer cell lines^11^.

This paper is structured as follows: at first, we present the problem and previous work, and then we present novel approximations based on our work using symbolic regression and analytic continued fractions. That introduces a very simple formula that is based on a particular feature of the known optimal configurations so far found. Finally, we present our conclusions from this study.

## The Thomson problem

The basic Thomson problem is simple to state but difficult to solve constrained non-linear global optimization; find the equilibrium configuration of *n* identical charges subjected to be on a surface of a sphere. This means that for each value of *n* the task is to find the global optimum. This has proved to be a challenge in itself, the problem is used as a classical benchmark to evaluate the performance of optimization packages^12^.

Although its long history, the problem is far from being solved for an arbitrary value of *n*, and it has the ongoing interest of chemists, physicists and mathematicians. In fact, it has a renewed interest lately since it is a special case of one of the “Eighteen unsolved mathematical challenges” proposed for the 21st Century by Steve Smale^13^.

This problem was posed in 1904 by J.J. Thomson and, in some sense, it has become a classical problem of ongoing interest both in Mathematics and Physics. His interest was to solve which is the 3-dimensional configuration of *n* electrons that minimizes the electrostatic potential if they are constrained to be on a surface of a unit sphere. The electrons repel each other with a force that is given by Coulomb’s Law.

Part of the renewed interest also lies in the generalizations and some of the present open conjectures. For instance, if *p*(*a*) and *p*(*b*) are the positions of two points on the sphere where two electrons *a* and *b* are located, and if we assume the Riesz potential, the energy of a set *S* of $|S|=n$ “electrons” will have potential energy given by

1 $$\begin{aligned} E(n) = \sum _{a,b \in S} \frac{1}{|p(a)-p(b)|^{s}} \end{aligned}$$

where $s>0$ and the case $s=1$ corresponds to the Coulomb potential; thus, in that case, we have the Thomson problem. It has been conjectured^14^ that for the minimum configuration potential, the Riesz potential, the value of the energy is given by:

2 $$\begin{aligned} E(n,s) = \frac{(\sqrt{3}/2)^{s/2} \, \xi _{\Lambda _2}(s)}{(4\pi )^{s/2}} n^{1+s/2} + V_s n^2 + o(n^2) \end{aligned}$$

where

3 $$\begin{aligned} V_s=\frac{2^{1-s}}{2-s}, \end{aligned}$$

$\Lambda _2$ is the regular planar triangular lattice generated by basis vectors (1, 0) and $(1/2, \sqrt{3}/2)$, and $\xi _{\Lambda _2}(s)$ is the Epstein-zeta function^15^ for the lattice $\Lambda _2$^14^ (see also^16,17^ for other seminal papers).

Having presented the relevance and interest of this problem, we now look at the history of the Thomson problem and how researchers have tried to obtain an analytical expression from existing data.

### Configurations with energy lower than the trend

Glasser and Every suggested that “minimum energy accurately follows a simple half-integral power law in 1/*n*”^18^. They first noticed how *“it is striking how close the configuration energies lie to a smooth curve. On the scale that has been used, scatter in the energies can barely be discerned”*. In fact, a number of optimal configurations seem to be characterized by a “significant lower (energy value) than the neighbours”. In^19^, the authors comment that the configurations for $n=12, 32, 72, 122, 132,$ and 192 have all in common the fact that they have icosahedral symmetry, and that “the icosahedral structures for $n=212, 272, 282,$ and 312 also have icosahedral symmetry and have low energy”. While they argue that this has been predicted, they also point out that the cases of $n=42, 92,$ and 162 are also icosahedral structures, but these have high energies relative to the trend of the equation we are fitting. D.J. Wales suggested that the energy lies at the tail of the distribution for the systems with high symmetry^20,21^. This explains that the structures with higher symmetry measures tend to have either high or low energy minima; hence the icosahedral structure was found to exhibit both high and low energy for the aforementioned cases.

While the question is outside the scope of this work, we point out, however, that the integer sequence **12**, 32, **42**, 72, **92**, 122, 132, **162**, 192, 212, **252**, 272,282, 312, **362**, 372, 392, 432, 482, **492**, 522, 572, 612, 632, **642**, 672, 732, 752, 762, 792, 812, $\ldots$ corresponds to the number of vertices of Goldberg-Casper-Klug pseudo-icosahedra in the curated Online Encyclopedia of Integer Sequences (OEIS) *A*071336. We underline those values of *n*, which were discussed before by Glasser and Every^19^. We also note that the numbers we write in boldface belong to another sequence curated at the OEIS (*A*005901), and it is one corresponding to the “Number of points on surface of cuboctahedron (or icosahedron): $a(0) = 1;$ for $n > 0,\, a(n) = 10 \, n^2 + 2$. Also coordination sequence for f.c.c. or $A_3$ or $D_3$ lattice.” Since the sequence *A*071336 contains the one defined by their recurrence relation of *A*005901, it may then be an interesting open problem to determine if other icosahedral configurations with “avoided” low energy values are exactly those defined by that specific subsequence of the number of vertices of Goldberg-Casper-Klug pseudo-icosahedra or if others exist. Very recently, Timothy Michaels used equidistributed icosahedral configurations based on combining the (*m*, *n*) icosahedral nodes of Casper and Klug and an azimuthal projection method to define a low deformation equal area mapping^22^. This allowed him to analyze the Riesz potential energies numerically up to $n < 50000$. The OEIS seems to be an interesting tool to analyze sequences of values of *n* on which we observed some property, and we introduced it here since it will be again used later in the manuscript as an exploratory tool.

### Approximations to the energy in Thomson problem as a function of the number of electrons

We briefly review here how several approximations have been given; generally, all of them are linked to an extension on the availability of conjectured or proved exact solutions for increasingly larger values of *n*. We will start with the already cited work of Glasser and Every and their derivation of a functional form.

#### Glasser and Every^18^

There has been huge progress in this problem in the last thirty years. Before 1992, optimal configurations for the problem were known only for $n\le 50$. In^18^, Glasser and Every finally extended the number of problems solved for up to 101 electrons. They observe that the values of the *“minimum energy accurately follows a simple half-integral power law in* 1/*n”*. They fit a function to the data by using the known minimum energies for all cases with $n\le 50$ and another 20 optimal configurations with $51 \le n \le 101$.

They propose a functional form for the energy as

4 $$\begin{aligned} E(n) = \frac{n^2}{2} \left( 1 + \frac{a}{n^{\alpha }} + \frac{b}{n^{\beta }} + \cdots \right) \end{aligned}$$

with $0< \alpha< \beta < \ldots \,$. Using only the values of the energies for $n=70$ ($E(70)=2127.100902$) and $n=80$ ($E(80)=2805.355876$), and leaving the first term of the expansion, they fit the free parameters as follows: $\alpha = 0.496$ and $a = -1.084$, concluding from the good fit to the first configurations with $n\le 30$ that $\alpha$ should be equal to 1/2 instead. Then they observe the need to include the second term of the expansion and, after another round of fitting and approximation of an exponent to a ratio of two integers they ended, finally, setting for a formula of the form:

5 $$\begin{aligned} E(n) = \frac{n^2}{2} \left( 1 - \frac{1.1028 }{n^{1/2}} + \frac{0.096}{n^{3/2}} \cdots \right) . \end{aligned}$$

A note added in proof indicates a better fitting with $a =-1.1039$ and $b = 0.105$ for $n\le 65$, so we will call the *“Glasser and Every model”* the one coming from the use of the formula with these values.

#### Morris, Deaven, Ho^19^

In 1996, Morris, Deaven and Ho presented results of what we consider a memetic algorithm^23,24^, a population-based strategy for finding the minimum energy configurations that use conjugate gradient optimization as a local search strategy. Not only have they been able to independently reproduce all the known optimal results for $10<n<132$, but they have been able to provide optimal configurations up to $n \le 200$. They use the same formula employed by the Glasser and Every model but with slightly different values of the parameters (i.e. $a = - 1.104616 \pm 0.000 01$ and $b=0.1376 \pm 0.001$), concluding that the fitted formula for the new information available still is in reasonable agreement with the previous fits and calculations by Every and Glasser.

## Approximations using symbolic and analytic continued fraction regression

Given the reported successes of symbolic regression in other problem domains, we first aimed at finding a fit to all the existing data that Morris, Deaven, Ho used. 

### An initial model of interest found with symbolic regression

Again, using all results for $n \le 200$, and using the commercial package Eureqa (together with a post-optimization procedure based on non-linear programming), we have been able to find an approximation model for the total energy of the form:

6 $$\begin{aligned} E(n) {\approx } \frac{n^2}{2} \, k^{{-} \gamma (n)} \end{aligned}$$

where Eureqa obtained

7 $$\begin{aligned} \gamma (n) = {\frac{4}{11 \sqrt{n} - 6}}, \end{aligned}$$

and $k=11591/547$. Once again, the simplicity of this model for *E*(*n*), defined by five integer constants, is quite impressive.

A Puiseux series expansion at $n=\infty$ for $\gamma (n)$ reveals an interesting role of the powers of the integers 12 and 11 in this approximation since it has the following form:

$$\begin{aligned} \begin{aligned} \gamma (n) =&4 \frac{1}{11} n^{-1/2} + 2 \frac{12}{11^2} n + 1 \frac{12^2}{11^3} n^{-3/2} + 6 \frac{12^2}{11^4} n^2 + 3 \frac{12^3}{11^5} n^{-5/2} + 18 \frac{12^3}{11^6} n^3 + 9 \frac{12^4}{11^7} n^{-7/2} + 54 \frac{12^4}{11^8} n^4 + 27 \frac{12^5}{11^9} n^{-9/2} + O\left( n^{-5}\right) . \end{aligned} \end{aligned}$$

And a Puisaux series at $n=\infty$ of $k^{ - \gamma (n)}$ is given by:

8 $$\begin{aligned} k^{ - \gamma (n)} \, = 1 - \frac{4}{11} \, log(k) n^{-1/2} + \frac{8 \, (log(k) - 3) \, log(k)}{121} n^{-1} + O(n^{-3/2}) \end{aligned}$$

with *log*(*k*) being the natural logarithm of *k*. We can observe that the second term of the expansion, $- 4/11 \, log(k)=-1.11037651326\ldots \,$, is relatively close to Morris, Deaven and Ho’s estimation of $a=-1.104616$, but we note that their original proposal did not include a term in $n^{-1}$. By comparison, the Puisaux series expansion of our model of Eq. (6) explains why perhaps a better approximation was not found until now.

#### Behaviour of the residual error and the relative error of the model of equation (6) for $n \le 200$

**Figure 1:**
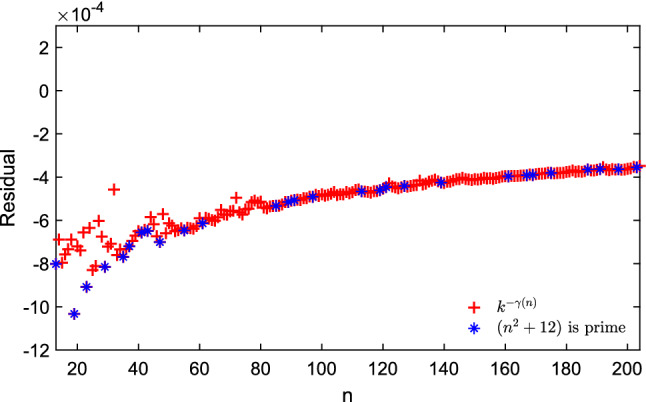
The difference between the exact value of the normalized energy and the value given by approximation of $k^{-\gamma (n)}$ in Eq. (7) for $13 \le n \le 204$ and highlighted the *n* where $n^2 + 12$ is a prime.

This motivated us to look at the behavior of both the residual error and the relative error for the values that we have used to find this approximation (i.e. $2 \le n \le 200$). For simplicity, we will refer to the normalized energy, i.e. $E_n(n) = 2 \, E(n) / n^2$. The difference between the exact value of the normalized energy and the one provided by our model for $n=2$ is $0.25 - k^{ - \gamma (2)} = {-} 0.028561067$. Figure 1 shows the difference between the exact value of the normalized energy and the value given by $k^{ - \gamma (n)}$ for $13 \le n \le 204$. For the configuration with the largest number of electrons in the figure, the difference is $0.922699961 - k^{ - \gamma (204)} =$ $\, {3.4829385 \times 10^{-4}}$.

### Approximations with continued fraction regression

We have then used symbolic regression to find a more fitting function than Eq. (7) to see if we can find a better approximation of very low complexity using the software Eureqa on all the known conjectured or proven optimal solutions to Thomson problems that are currently known. Unfortunately, no better solution than Eqs. (6) and (7) was found. We then turned our attention to a new method we have recently developed based on analytic continued fractions.

The analytic continued fraction regression method was introduced in^25^ as a general approach to model an unknown target function of multivariate independent variables, ${\textbf{x}}$, as a generalized continued fraction. For a multivariate unknown target function $f : \mathbb {R}^d \rightarrow \mathbb {R}$, we then write:

9 $$\begin{aligned} f({\textbf{x}}) = b_0({\textbf{x}})\, + \, \frac{a_1({\textbf{x}})}{b_1({\textbf{x}}) + \frac{a_2({\textbf{x}})}{b_2({\textbf{x}}) + \frac{a_3({\textbf{x}})}{b_3({\textbf{x}}) + \ddots }}} \end{aligned}$$

where $a_i({\textbf{x}}), b_i({\textbf{x}}) \in \mathbb {R}$ for all integer ($i\ge 1$, respectively $i\ge 0$). For each function $a_i({\textbf{x}}): \mathbb {R}^d \rightarrow \mathbb {R}$ is associated with a *d*-dimensional vector $\mathbf {a_i} \in \mathbb {R}^d$ and a constant $\alpha _i \in \mathbb {R}$:

10 $$\begin{aligned} a_i({\textbf{x}}) = \mathbf {a_i}^{\textrm{T}} {\textbf{x}} + \alpha _i \end{aligned}$$

Analogously, each function $b_i({\textbf{x}}) : \mathbb {R}^d \rightarrow \mathbb {R}$ is associated with a vector $\mathbf {b_i} \in \mathbb {R}^d$ and a constant $\beta _i \in \mathbb {R}$:

11 $$\begin{aligned} b_i({\textbf{x}}) = \mathbf {b_i}^{\textrm{T}} {\textbf{x}} + \beta _i. \end{aligned}$$

As an example, given a function *f*(*x*) we can use Mathematica to find its analytic continued fraction expansion. For instance, given $f(x)=(\sqrt{4x+1}-1)/2$ we can approximate it with arbitrarily precision. Truncating the expansion at $depth=6$ we have:

$$\begin{aligned} \frac{\sqrt{4x +1}-1}{2} \approx x + \frac{-x^2}{1+\frac{2x}{1+\frac{\frac{x}{2}}{1+\frac{\frac{3x}{2}}{1+\frac{\frac{2x}{3}}{1+\frac{\frac{4x}{3}}{1}}}}}} \end{aligned}$$

This function and a continued fraction expansion is discussed in^26^ (this expansion we present was provided by Mathematica and it has $b_i(x,x^2) = 1, \forall i\ge 0$ and $b_0(x,x^2) = x$), but many others could have been used to illustrate the expansion. The authors of^26^ point to the interesting *“Handbook on continued fractions”* contributed by Annie Cuyt et al.^27^ in which many expansions of interests are which may be of interest for some of the readers.

For the application of these ideas for regression, the methodology proposed in^25^ implies that, given a dataset, we face both a combinatorial and a non-linear optimization problem to solve. The first one comes from the selection of the variables that need to be used in the expansion (in the example given, both *x* and $x^2$ are used). Associated to each of the models that need to be iteratively created, we need to estimate the associated coefficients via a non-linear optimization algorithm or heuristic. Since^25^ all the subsequent papers^28–30^ have been employing a memetic algorithm, i.e. a population-based search technique, is used as the method of choice to find both the variables and coefficients.

A Nelder-Mead (NM) based local search optimizes the current solutions at each generation in the memetic algorithm and several heuristics exist for the inclusion and rejection of variables in the models. Details of the method can be found in^29^. In^28^, the authors present results on 452 datasets of physical relevance; the method showed a very good performance, and in^29^ another comprehensive review of comparative performance against many state-of-the-art regression algorithms is also given on a large multivariate dataset used for testing.

#### A first approximation using continued fraction regression

We used a method based on multivariate regression using an analytic continued fraction representation of our target function of interest^25,28–31^. We looked at approximating *E*(*n*) as follows:

12 $$\begin{aligned} E(n) \approx \frac{n^2}{2} \, e^{g(n)} \end{aligned}$$

We executed our memetic algorithm based continued fraction regression (CFR) method to approximate *g*(*n*) for $depths=\{0,1,2\}$^29^. The CFR method uses a continued fraction-based representation of the unknown function that we are aiming to fit.

We found the following $depth=2$ model for *g*(*n*):

13 $$\begin{aligned} g(n)={\left( a\, u +\frac{b\, u+ c}{(d\, u + e)+\frac{f\,u + g}{h\, u + i}}\right) } \end{aligned}$$

as the best approximation (with a with $MSE={6.1080 \times 10^{-8}}$) for normalized energy where $u=n^{-1/2}$ and the coefficients are: *a* = − 0.880367889446965535, *b* = 0.793386942751073909, *c* = 0.184694236906771669, *d* = 1.27484122748516482, *e* = − 0.488981361176515139, *f* = 0.0528983408465706698, *g* = 0.298731221071015351, *h* = − 0.341376009238492262, *i* = − 0.000516314596688742609.

All the residuals presented in this paper are plotted on the same range, between $-12 \times 10^{-4}$ and $2 \times 10^{-4}$ and for $13< n < 204$. In particular, this helps to compare the results with the previous one given by Eqs. (6) and (7).

**Figure 2:**
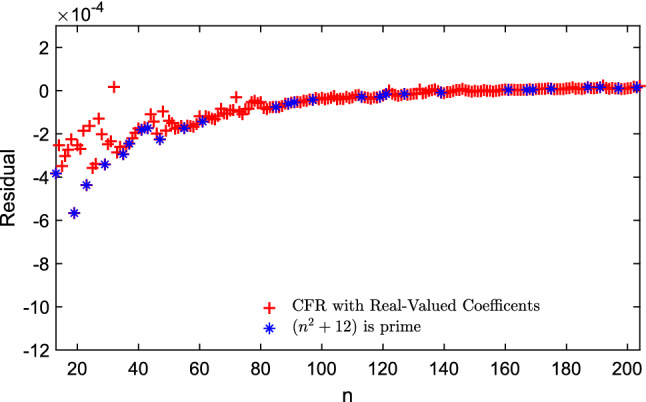
The difference between the exact value of normalized energy and the value approximated as $e^{g(n)}$, where *g*(*n*) is given by the Continued Fraction regression approximation in Eq. (13) for fitting the experimental data for $13 \le n \le 204$ and highlighted the *n* where $n^2 + 12$ is prime.

From Figs. 1 and 2 we can observe that for increasing values of *n*, we now have an approximation that converges faster to a desired zero residual, with noticeable differences already in the range of $n\le 204$ in Fig. 2.

#### An approximation using $n^{-1/2}, n^{-1}$ and, $n^{-3/2}$ as independent variables

**Figure 3:**
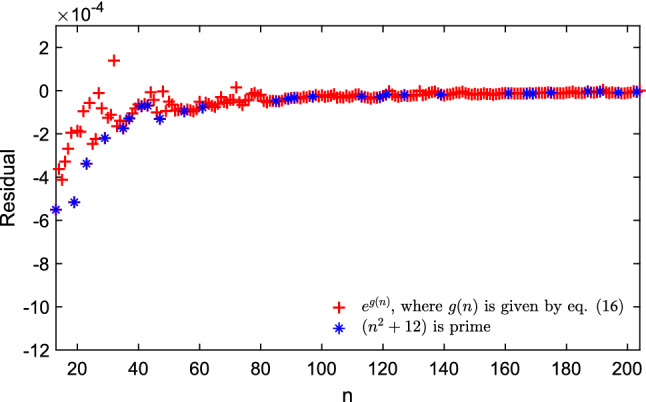
The difference between the exact value of the normalized energy and the value given as $e^{g(n)}$, where *g*(*n*) is given by the model in Eq. (15) for fitting the experimental data for $13 \le n \le 204$ and highlighted the *n* where $n^2 + 12$ is prime.

We then look at the possibilities that we can have by explicitly employing $n^{-1/2}, n^{-1}$ and, $n^{-3/2}$ as independent variables since our memetic algorithm for analytic continued fraction regression can handle well multi-dimensional problems. In this case, we have managed to obtain a new approximation for *E*(*n*) of the form:

14 $$\begin{aligned} E(n) \approx \frac{n^2}{2} \, e^{g(n)} \end{aligned}$$

where *g*(*n*) is now an explicit function of these variables:

15 $$\begin{aligned} g(n) = - n^{-1/2} + \frac{7 n^{-1/2} + 35 n^{-1} - 14 n^{-3/2}}{58 n^{-1/2} - 67}, \end{aligned}$$

here, the mean square error of normalized energy (i.e. $2 E(n) / n^2$) is ${8.4161 \times 10^{-8}}$ (difference between the exact normalized energy data and the approximation are showed in Fig. 3 for $13 \le n \le 204$ with highlighting the *n* where $n^2 + 12$ is prime). Most notably, the Puisaux series at $n=\infty$ of $e^{g(n)}$ is given by:

16 $$\begin{aligned} e^{g(n)} = 1 - \frac{74}{67} n^{-1/2} - \frac{13}{4489} n{^{-1}} + \frac{117974}{902289} n^{-3/2} + O(n^{-2}), \end{aligned}$$

so the second term of the expansion is $-74/67 = - 1.10447761\ldots \,$, again very close to Morris, Deaven and Ho’s previous estimate of $-1.104616$.

### A new solution with ratios of integers as coefficients

With Eq. (13), we used Mathematica to simplify it as a ratio of two polynomials. Then the coefficients were optimized further and presented as a ratio of two integers, as it is common in many analytic continued fractions. We used a high-precision non-linear optimization routine of Matlab. Consequently, we obtained this other expression with a reduced MSE value (${5.5549 \times 10^{-8}}$) to calculate the normalized energy:

17 $$\begin{aligned} { g(n) = \left( \frac{ \frac{12}{20467}\, n + \frac{4421}{1523}\, \sqrt{n} - \frac{998}{495\, \sqrt{n}} + \frac{8675}{6156} }{ -\frac{8998}{3401}\, n + \frac{1271}{3564}\, \sqrt{n} + \frac{9641}{5261} } \right) } \end{aligned}$$

**Figure 4:**
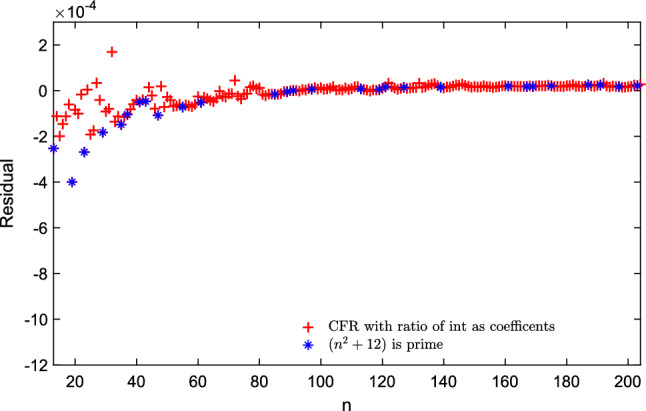
The difference between the exact value of normalized energy and the value approximated as $e^{g(n)}$, where *g*(*n*) is given by the Continued Fraction regression model with ratio of integer coefficients in Eq. (17) for fitting the experimental data for $13 \le n \le 204$ and highlighted the *n* where $n^2 + 12$ is prime.

We showed the residual of the continued fraction regression model with only ratio of integer coefficients for approximating the Thomson values in the range of $n\le 204$ in Fig. 4. This is a remarkably good approximation in all the ranges, even for the case of two electrons (giving a value of 0.2504976, when the exact value of the normalized energy is 0.25). For the largest configuration in the dataset (with $n=4352$ electrons^32,33^, lowest minima located for the Thomson problem: http://www.junlilab.org/database/TP2.html), the approximation is 0.98313334728 for the known solution of value equal to 0.983244295.

**Figure 5:**
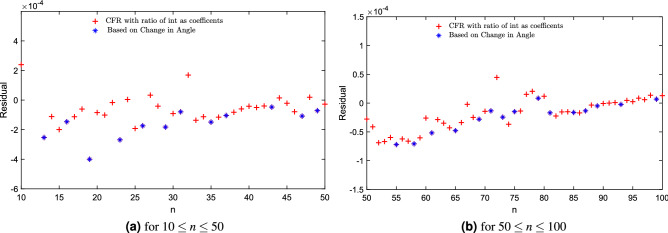
The difference between the exact value of normalized energy and the value approximated as $e^{g(n)}$, where *g*(*n*) is given by the Continued Fraction regression model with ratio of integer coefficients in Eq. (17). The plot is shown to fit the experimental data for (a) $10 \le n \le 50$, (b) $50 \le n \le 100$, and highlights the *n* for an angle-based new sequence of integers.

## A simple equation based on minimum angles

We have shown before that, at least for optimal configurations with small *n*, all approximations seem to err somewhat more than the trend when $n^2+12$ is prime. We have not yet found an explanation for this fact. We then turned our attention to some other proven and putative optimal known solutions for $n\le 200$^32^ (Global Minima for the Thomson Problem: https://www-wales.ch.cam.ac.uk/~wales/CCD/Thomson/table.html, https://www-wales.ch.cam.ac.uk/~wales/CCD/Thomson/xyz/) and we found a pattern worth exploring further.

### A new sequence of integers

In fact, a new sequence of integers can be defined, which at the time of preparing this manuscript does not seem to be currently listed in the Online Encyclopedia of Integer Sequences, while other sequences related to the Thomson problem are. It is: $11, 13, 16, 19, 23, 26, 29, 31,\ldots \,$. This sequence is defined in the following way. Let $\alpha (n)$ is the smallest angle, measured in radians, which is subtended by the vectors associated with the nearest charge pair in the optimal configuration for *n* electrons. We then say that an integer *n* is in this sequence if and only if $\alpha (n) < \alpha (n+1)$. This means that in the optimal configuration for *n* electrons there is at least one pair which is near than the nearest pair in a configuration with $n+1$ electrons.

We have observed that there was an overlap between this integer sequence and those *n* such that $n^2 + 12$ is prime (e.g. 19, 23, 26, 29) (OEIS *A*114275). Motivated by this fact, we looked at the possibility that $\alpha (n)$, together with the inverse of the square root of the number of electrons, could provide a low complexity but good approximation formula for the energy of the optimal configuration.

Using symbolic regression (in this case the commercial package TuringBot), we have also found the following simple formula, which is a good approximation for *E*(*n*):

18 $$\begin{aligned} E(n) \approx \frac{n^2}{2} \left( \frac{\sqrt{n} -1}{\sqrt{n} + \frac{\alpha (n) + \pi }{30} } \right) . \end{aligned}$$

**Figure 6:**
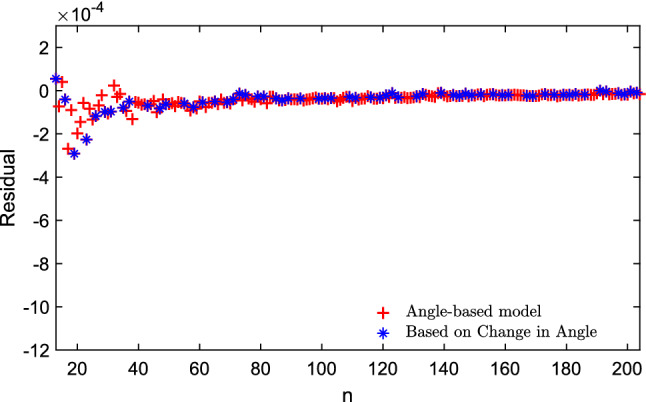
The difference between the exact value of normalized energy and the value approximated by the Angle-based model in Eq. (18). The plot is shown to fit the available data for $13 \le n \le 204$ and highlights the *n* for an angle-based new sequence of integers.

The formula was obtained by only accepting integer coefficients in the expressions generated by the symbolic regression method TuringBot. The constant $\pi$ was incorporated ad hoc in the formula after interpreting that ‘$\alpha (n) + 3$’ may actually be representing an angle measured in radians ‘$\alpha (n) + \pi$’. We noted that the ‘3’ in that formula is a consequence of the symbolic regression approach (that searched for integer coefficients), and we present this formula after observing that the MSE improved with the substitution.

This is very interesting, in the limit for $\alpha (n)$ tending to zero, we have that

19 $$\begin{aligned} \lim _{\alpha (n) \rightarrow 0} E(n) \approx \frac{n^2}{2} \left( \frac{ 30 \, (\sqrt{n} - 1)}{ 30 \, \sqrt{n} + \pi } \right) \end{aligned}$$

and if we take a look at the series expansion at $n=\infty$ we have

20 $$\begin{aligned} \lim _{\alpha (n) \rightarrow 0} E(n) \approx \frac{n^2}{2} \left[ 1 + \left( -1 - \frac{\pi }{30} \right) \, n^{-1/2} + \cdots \right] \end{aligned}$$

an expression that includes the transcendental number

21 $$\begin{aligned} -1 - \frac{\pi }{30} = -1.10471975511965977\ldots \end{aligned}$$

again, a result that is in reasonable agreement with the previous estimate by Morris, Deaven and Ho $( -1.104616\ldots \,)$ which, it is useful to recall, was obtained by analyzing only electron configurations with $n \le 200$. Using a non-linear optimization approach and all the data we obtained $-1 - \pi /29.96627907927524\ldots \approx -1.104837\ldots \,$. When we used the 254 configurations not used by Morris, Deaven and Ho, i.e. those with $200 \le n \le 4352$, we obtained $-1-\pi /30.0270165553\ldots \,$, leading to a value of $-1.10462553440167\ldots \,$.

**Figure 7:**
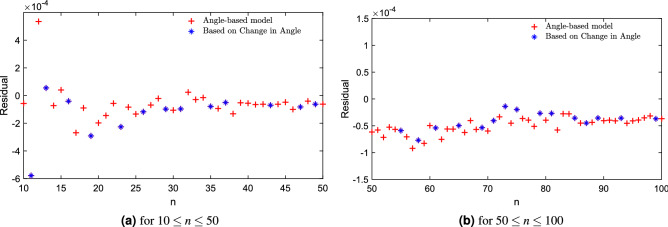
The difference between the exact value of normalized energy and the value approximated by the model with including minimal angles of Eq. (18). The plot is shown to fit the experimental data for (**a**) $10 \le n \le 50$, (**b**) $50 \le n \le 100$, and highlights the *n* for an angle-based new sequence of integers defined in this work.

We present our previous model in Eq. (17) which highlights the *n* for the angle-based new sequence (11, 13, 16, 19, 23, 26, 29, 31,$\ldots \,$ here defined for the first time) of $10 \le n \le 50$ in Fig. 5a and $50 \le n \le 100$ in Fig. 5b. We present the residual calculated between exact and approximation by Eq. (18) in Fig. 6. Also, Fig. 7a ($10 \le n \le 50$ ) and 7b ($50 \le n \le 100$ ) show that, for the new integer sequence, the points in this sequence do not look as outliers of the general trend, indicating that the geometric information introduced by just including the minimum angle between pairs of electrons leads to a model of low complexity and also good approximation capability.

As Supplementary Information, we have provided the approximations by the models presented in this work along with the MSE score for the prediction of normalized energy in Table S1 and Energy of Thomson in Table S2 for the reader’s convenience.

## Conclusions

In this paper, we have shown that the minimum potential energy of the Thomson problem, *E*(*n*), can be well approximated by a formula for the form: $E(n) = (n^2/2) \, e^{g(n)}$ where *g*(*n*) is obtained using a truncated analytic continued fraction expansion. The methodology used here is very general and could eventually be used for other cases such as Reisz potential and other variants of this problem of interest^35–36^. Investigation of the sequence of values of *n* for which the residual error of our approximations was a bit higher than the trend lead to the introduction of a new sequence of integers. This, in turn, suggested that perhaps another expression could be found involved $\alpha (n)$ is the smallest angle, measured in radians, which is subtended by the vectors associated with the nearest charge pair in the optimal configuration for *n* electrons. These values are only known after a very hard non-linear optimization is solved. Future work involves finding an approximation of $\alpha (n)$ using only the value of *n* which could be potentially something of interest for building constructive heuristics for the challenging non-linear optimization problem for the general case of *n* electrons.

## Supplementary Information

Supplementary Information.

## Data Availability

The datasets generated during and/or analyzed during the current study are available in the public domain. The Global Minima for the Thomson Problem for $n\le 400$ are available^32^ at: https://www-wales.ch.cam.ac.uk/~wales/CCD/Thomson/table.html and^37^ https://en.wikipedia.org/wiki/Thomson_problem. The files containing (*x*, *y*, *z*) coordinate values are available^32^ at: https://www-wales.ch.cam.ac.uk/~wales/CCD/Thomson/xyz/. The lowest found minima for the Thomson problem for some larger values in the range of $400 \le n\le 4352$ (and associated (*x*, *y*, *z*) coordinates of the configurations) are available^33^ at: http://www.junlilab.org/database/TP2.html.
