# Supplementary material for: Continued fractions and the Thomson problem
Source: Sci Rep. 2023 May 4;13:7272. doi: 10.1038/s41598-023-33744-5 (PMC10160075; doi:10.1038/s41598-023-33744-5)
Supplement: Supplementary file 1 — Supplementary Information. [file 41598_2023_33744_MOESM1_ESM.pdf]

Supplementary Information: Continued fractions and the Thomson Problem

Pablo Moscato[1,*], Mohammad Nazmul Haque[1], and Anna Moscato[2]

[1]School of Information and Physical Sciences, The University of Newcastle, Callaghan, NSW 2308, Australia
[2]Australian National University, Canberra, ACT 2601, Australia
[*]Pablo.Moscato@newcastle.edu.au

Supplementary Information

Approximation of Normalised Energy by the models

In this work we found five different models to approximate the normalised energy, $E_n(n) \equiv 2E(n)/n^2$. The detail of the mathematical models are in the original article. We are showing the estimations of $E_n \equiv e^{g(n)}$ by those models in Table S1 for the convenience of the reader.

Table S1. The approximations to the Normalised Energy ($E_n(n) \equiv 2E(n)/n^2$) in Thomson problem presented by the models in this contribution.

n	$\alpha(n)$	$E_n(n)$	$k^{\gamma(n)}$ by eq. (7)	$e^{g(n)}$ by eq. (13)	$e^{g(n)}$ by eq. (15)	$e^{g(n)}$ by eq. (17)	$\left(\frac{\sqrt{n}-1}{\sqrt{n}+\frac{\alpha(n)+\pi}{30}}\right)$ by eq. (19)
2	3.141592654	0.25	0.27856106702663663	0.25014341764749354	0.2514529190530735	0.25049761444354285	0.25511211068208567
3	2.094395102	0.38490018	0.3922852349626302	0.3824439704825541	0.380730384964359	0.3823779263199175	0.38395943225162776
4	1.910629385	0.459279327	0.46608658711059364	0.46198223366427094	0.46009799044725697	0.461826258584616	0.4611679518372735
5	1.570796327	0.51797532	0.5185135477390602	0.5166743129901579	0.515080277896092	0.5165404095031063	0.5165030239834058
6	1.570796327	0.554737854	0.5581262088954253	0.5573291645292766	0.5560610179120955	0.5572441561243662	0.55609098933808
7	1.256637061	0.589917445	0.5893847930651213	0.589118476942821	0.588130448335219	0.5890833405713323	0.5893766989833349
8	1.251296354	0.614852746	0.6148523462222427	0.6148763353736332	0.6141142446200524	0.6148848350266342	0.6146269461965861
9	1.207593309	0.63604905	0.6361151894757173	0.636306561944709	0.635723567137956	0.6363511788176461	0.6359355344495551
10	1.1343877	0.654338989	0.6542129674446275	0.6545047945109429	0.6540634199973074	0.6545785698637022	0.6542819219908367
11	1.021480486	0.671015711	0.6698585772223493	0.670212605062828	0.6698835478148955	0.6703096115942621	0.6704372528127572
12	1.107148717	0.682850737	0.6835590723035984	0.6839525844239356	0.6837130291818566	0.6840679378351666	0.6833856458686207
13	0.912565852	0.696487936	0.6956861381540381	0.6961052260995989	0.6959373538832212	0.6962349568154772	0.6965429774907407
14	0.922687346	0.707207789	0.7065192040724106	0.7069551396924991	0.7068449488000815	0.7070960381556047	0.7071351726345607
15	0.859136178	0.717068837	0.7162729028932248	0.7167200983752208	0.7166565075197677	0.7168695721090201	0.7171090441139549
16	0.85409817	0.725872307	0.7251151710273426	0.7255699906091071	0.7255441649411889	0.725725943432876	0.7258316752962337
17	0.874550799	0.733912836	0.7331795355262738	0.7336395824055874	0.7336444275615138	0.733800316163907	0.7336439852272225
18	0.829632142	0.741262144	0.7405736709508808	0.7410373483955369	0.7410671291177698	0.7412014843473439	0.7411724989603975
19	0.783817347	0.748418103	0.747385493411757	0.7478517287339553	0.7479017795063249	0.7480181450985554	0.7481267282747613
20	0.804480122	0.754407842	0.7536875873207556	0.7541556550419862	0.7542221588599979	0.7543234374607851	0.7542101545382623
21	0.773491939	0.760279467	0.7595404780999885	0.760009884860218	0.7600897027348895	0.7601782870526093	0.7601349486912093
22	0.755762586	0.765650976	0.764995090134476	0.7654654987765561	0.765556037282121	0.7656339112015059	0.7655943880138534
23	0.723981994	0.771002611	0.7700946191949836	0.770565798123452	0.7706649056945951	0.7707337229231777	0.7707766659542293
24	0.734178063	0.775510674	0.7748759772639646	0.7753477663500215	0.7754536514780948	0.7755147972313474	0.7754268564150752
25	0.691324989	0.780200833	0.7793709205229753	0.7798432079806075	0.7799543742226913	0.7800090139891842	0.7800671133261865
26	0.677940327	0.784418125	0.7836069394375366	0.7840796460550684	0.7841948400506195	0.7842439584306219	0.784300609450285
27	0.697089572	0.788210192	0.7876079680292231	0.7880810363791759	0.7881992060050796	0.7882436378598053	0.788141794096687
28	0.660148882	0.792070261	0.7913949541848028	0.7918683412302161	0.791988601714165	0.7920290573122309	0.7920491985379299
29	0.635148529	0.795801284	0.794986322063187	0.7954599940963866	0.7955816004196359	0.7956186858628151	0.7957031581601519
30	0.644766305	0.79911988	0.7983983499186026	0.7988722791098934	0.7989946034124553	0.7990288373230985	0.7990140949767068
31	0.634775055	0.802353461	0.8016454810323634	0.8021196430939287	0.8022421560828964	0.8022739833114726	0.8022568561154608
32	0.652358139	0.805197802	0.8047405813096576	0.8052149539334866	0.8053372095133513	0.8053670124559001	0.8052217121901827
33	0.588109533	0.808455569	0.807695154024227	0.8081697158573585	0.8082913383678153	0.8083194463550989	0.8084264379794255
34	0.580731094	0.811254071	0.8105195198866946	0.8109942498787268	0.811114923453621	0.8111416205758905	0.8112389727066324
35	0.577711758	0.813991629	0.8132229688641212	0.8136978458714721	0.8138173055317302	0.8138428371876723	0.8139128672549937
36	0.579959056	0.816546927	0.8158138888420227	0.8162888914078866	0.8164069155785442	0.816431493978556	0.8164529343683438
37	0.56430746	0.819019558	0.8182998751900663	0.8187749814431222	0.8188913856450991	0.8189151944534849	0.8189692159020906
38	0.58008623	0.821382969	0.820687824492577	0.8211630121244555	0.8212776436394464	0.8213008419043661	0.8212512791016698

Continued on next page

Table S1 – *Continued from previous page*

**n**	$\alpha(n)$	$E_n(n)$	$k^{\gamma(n)}$ by eq. (7)	$e^{g(n)}$ by eq. (13)	$e^{g(n)}$ by eq. (15)	$e^{g(n)}$ by eq. (17)	$\left(\frac{\sqrt{n}-1}{\sqrt{n}+\frac{\alpha(n)+\pi}{30}}\right)$ by eq. (19)
39	0.559429509	0.823654187	0.8229840150789409	0.8234592613724185	0.8235719947170688	0.8235947202088449	0.8236019238553489
40	0.557045422	0.825844099	0.8251941764957305	0.8256694583840931	0.8257801914597451	0.8258025635157282	0.8257885790299466
41	0.550264431	0.82797947	0.8273235496712046	0.8277988438152227	0.8279074946236288	0.8279296165799456	0.8279145156628636
42	0.545323649	0.83002053	0.8293769392106903	0.8298522220839177	0.8299587259187013	0.8299806871948715	0.8299577860497233
43	0.538726737	0.832007406	0.8313587590108144	0.8318340069869459	0.8319383140262657	0.8319601919171239	0.831937448337754
44	0.545548145	0.83385771	0.8332730721783409	0.8337482616165066	0.8338503348550939	0.8338721960750958	0.8337952853433015
45	0.527215189	0.835741631	0.8351236260752957	0.8355987334006835	0.835698546869784	0.835720448887178	0.8356937265147584
46	0.519938255	0.837587064	0.8369138831782593	0.8373888849565162	0.837486422188752	0.837508414380884	0.8374870928897509
47	0.502410384	0.839347461	0.8386470483300787	0.8391219213346788	0.8392171740378087	0.8392392986937391	0.8392651197080176
48	0.518182258	0.840897097	0.8403260928720003	0.8408008141442739	0.8408937810535727	0.8409160742459986	0.840856546336176
49	0.495433538	0.84261323	0.8419537760696206	0.8424283229714767	0.842519008855207	0.842541501200218	0.8425505784684342
50	0.501108446	0.844145852	0.8435326641841067	0.8440070144437047	0.8440954292400972	0.844118146560429	0.8440841508689064
51	0.491579154	0.845689573	0.845065147488511	0.8455392792392749	0.8456254373067175	0.8456484012117804	0.8456316069678763
52	0.48293345	0.847203376	0.8465534554858049	0.8470273472992517	0.8471112667641344	0.8471344951581017	0.8471317849379703
53	0.473626491	0.848645276	0.8479996705489752	0.8484733014618708	0.8485550036508281	0.8485785111784055	0.8485927220079784
54	0.471756582	0.850042164	0.8494057401729564	0.8498790897093177	0.849958598654548	0.8499823970926443	0.8499855609414767
55	0.46451825	0.851419981	0.8507734880023148	0.8512465361907713	0.8513238781987372	0.851347976801078	0.8513609452555368
56	0.465704108	0.852739123	0.8521046237766676	0.8525773511636755	0.8526525544388663	0.8526769602395976	0.8526685499678767
57	0.466045503	0.854037075	0.8534007523171573	0.8538731399765274	0.8539462342931302	0.853970952374617	0.8539450325405827
58	0.456495041	0.855302171	0.8546633816613625	0.8551354112005345	0.8552064276158509	0.8552314613451593	0.8552251378603924
59	0.456756909	0.856520158	0.8558939304403946	0.8563655840038534	0.8564345546081396	0.8564599058460834	0.8564373850132637
60	0.453045977	0.857683556	0.857093734580215	0.8575649948504114	0.8576319525485402	0.8576576218346514	0.8576339343853094
61	0.443161129	0.85887763	0.8582640533991301	0.8587349035952342	0.85879988191619	0.858825868632533	0.858823670711396
62	0.451689298	0.85999449	0.8594060751647271	0.8598764990395099	0.8599395319702567	0.8599658344866178	0.8599191479793741
63	0.440813525	0.86111347	0.8605209221659808	0.8609909040010912	0.8610520258418034	0.8610786416444695	0.8610574976427896
64	0.434936637	0.86220829	0.8616096553497434	0.862079179949617	0.8621384251876527	0.8621653509937041	0.8621520333326838
65	0.428073349	0.863274774	0.8626732785651491	0.8631423312497597	0.8631997344500933	0.863226966308896	0.8632251392233647
66	0.432227624	0.864298221	0.8637127424545228	0.8641813090511667	0.8642369047612843	0.8642644381446574	0.8642357345582381
67	0.431572387	0.865280775	0.8647289480250685	0.8651970148593469	0.8652508375268589	0.8652786674092109	0.86524039684068
68	0.426435315	0.866295286	0.865722749931832	0.8661903038179768	0.8662423877194225	0.866270508648992	0.8662385175051514
69	0.42126175	0.867268844	0.8666949594991191	0.8671619877297949	0.8672123669092969	0.8672407730714976	0.8672153588659386
70	0.423954129	0.86820445	0.8676463475046455	0.8681128378403377	0.8681615460569325	0.8681902313306812	0.8681447278794432
71	0.41543484	0.869133071	0.8685776467481234	0.8690435874062138	0.8690906580888251	0.8691196160966308	0.8690925004914921
72	0.42746095	0.869985027	0.8694895544237388	0.8699549340673555	0.8700004002765008	0.8700296244289983	0.8699516546054078
73	0.398108024	0.87094535	0.870382734313969	0.8708475420406849	0.8708914364361169	0.8709209199716523	0.8709315600377219
74	0.40083295	0.871830892	0.8712578188204275	0.8717220441508743	0.8717643989644492	0.8717941349842534	0.8717856900236377
75	0.396825722	0.872664778	0.8721154108458512	0.8725790437123035	0.8726198907254591	0.8726498722248837	0.8726450626032715
76	0.399429975	0.87350238	0.8729560855399557	0.8734191162749374	0.8734584868002252	0.8734887066964648	0.873465895365502

Continued on next page

Table S1 – *Continued from previous page*

n	$\alpha(n)$	$E_n(n)$	$k^{\gamma(n)}$ by eq. (7)	$e^{g(n)}$ by eq. (13)	$e^{g(n)}$ by eq. (15)	$e^{g(n)}$ by eq. (17)	$\left(\frac{\sqrt{n}-1}{\sqrt{n}+\frac{\alpha(n)+\pi}{30}}\right)$ by eq. (19)
77	0.406419914	0.874295885	0.8737803919206457	0.8742428112455963	0.8742807361117887	0.8743111872684627	0.8742563837471099
78	0.408867018	0.875097461	0.8745888543809608	0.875050653395	0.8750871629353394	0.875117838184267	0.8750463720064271
79	0.395072283	0.875900771	0.8753819740911541	0.875843144259974	0.875878268303187	0.8759091604636521	0.8758740286570776
80	0.397557423	0.876673711	0.8761602303044215	0.8766207634493322	0.8766545313130703	0.8766856332088403	0.8766341183122303
81	0.382085083	0.877464664	0.876924081574008	0.8773839698611563	0.877416410347568	0.8774477148219033	0.8774377486658615
82	0.387566912	0.878218226	0.8776739668887144	0.878133202818495	0.8781643442116643	0.8781958441405262	0.8781602797797954
83	0.377797357	0.878945707	0.8784103067331898	0.8788688831298627	0.8788987531948802	0.878930441498528	0.8789181095593263
84	0.375467045	0.879666985	0.8791335040788313	0.8795914140803547	0.8796200400638142	0.8796519097169576	0.8796392180366926
85	0.375201755	0.88037687	0.8798439453105905	0.8803011823586808	0.8803285909904183	0.8803606350310764	0.8803412349134919
86	0.375620201	0.881073988	0.880542001094532	0.8809985589249572	0.881024776420867	0.8810569879580672	0.8810289412789326
87	0.374484403	0.881754723	0.8812280271905681	0.8816838998236771	0.8817089518894635	0.8817413241099021	0.881709681454499
88	0.374994001	0.882417406	0.8819023652144182	0.8823575469459116	0.8823814587816441	0.8824139849554162	0.8823738105475986
89	0.369699044	0.883080171	0.8825653433524963	0.8830198287444398	0.8830426250498007	0.8830752985352964	0.8830446835309458
90	0.37053855	0.883726228	0.8832172770331301	0.8836710609052111	0.8836927658853324	0.8837255801333855	0.8836857347053134
91	0.368345801	0.884365101	0.8838584695572261	0.8843115469782513	0.8843321843500536	0.8843651329074222	0.8843255136954804
92	0.366969915	0.884993298	0.8844892126912451	0.8849415789708811	0.8849611719698312	0.8849942484820804	0.8849524808219102
93	0.362168686	0.885615525	0.8851097872251229	0.8855614379058743	0.8855800092930947	0.8856132075069431	0.8855797767692484
94	0.365678982	0.88621758	0.8857204634975577	0.8861713943469821	0.8861889664166482	0.8862222801818362	0.8861721910830431
95	0.361478702	0.886819208	0.8863215018908976	0.8867717088940529	0.8867883034810224	0.8868217267517532	0.8867783375003118
96	0.36104895	0.887403214	0.8869131532976845	0.8873626326498046	0.887378271137431	0.8874117979734304	0.8873638962358581
97	0.356913581	0.887986736	0.8874956595607533	0.8879444076601503	0.8879591109882362	0.8879927355554718	0.8879514613444511
98	0.356424582	0.888551117	0.8880692538886409	0.8885172673298253	0.8885310560026792	0.8885647725737773	0.8885193763099869
99	0.354030497	0.889121347	0.8886341612479243	0.8890814368149423	0.8890943309095032	0.8891281338638959	0.8890843488803254
100	0.354245493	0.889670127	0.8891905987339869	0.8896371333939678	0.8896491525679688	0.8896830363918032	0.8896332867325477
101	0.349260497	0.890224498	0.8897387759216016	0.8901845668185107	0.8901957303186568	0.8902296896044913	0.8901892874864233
102	0.34976316	0.890760586	0.890278895196616	0.890723939645208	0.8907342663153431	0.8907682957616575	0.8907212938663827
103	0.3474431	0.891287891	0.8908111520699318	0.8912554475498998	0.8912649558391444	0.8912990502496846	0.891253752537836
104	0.348308443	0.891804091	0.8913357354748845	0.8917792796251984	0.891787987596041	0.8918221418790185	0.8917694306210557
105	0.346305432	0.892335717	0.8918528280490522	0.8922956186624819	0.8923035439988078	0.8923377531659726	0.8922859968251117
106	0.34310174	0.892841687	0.8923626064014444	0.8928046414192632	0.8928118014343086	0.8928460605999111	0.8927987558293405
107	0.337317442	0.893345017	0.8928652413659638	0.8933065188728252	0.8933129305170445	0.8933472348967009	0.8933117624646376
108	0.337312652	0.893829477	0.8933608982419621	0.8938014164609462	0.8938070963297842	0.8938414412392588	0.893801388352282
109	0.333401708	0.894324565	0.8938497370226623	0.8942894943104865	0.8942944586520458	0.8943288395059622	0.8942953407278836
110	0.339913005	0.894801536	0.8943319126121634	0.8947709074545512	0.8947751721771494	0.8948095844876409	0.8947533389257482
111	0.3360664	0.89526714	0.8948075750316946	0.8952458060389	0.8952493867185095	0.8952838260938185	0.8952340951563741
112	0.337747247	0.895734197	0.8952768696157458	0.8957143355182253	0.8957172474057921	0.8957517095488269	0.8956930792810748
113	0.331220583	0.896205651	0.8957399371986527	0.8961766368428833	0.8961788948715204	0.8962133755783773	0.896168790714224
114	0.328746066	0.896663831	0.896196914292184	0.8966328466366201	0.8966344654286729	0.8966689605871307	0.896627151055907

Continued on next page

Table S1 – *Continued from previous page*

**n**	$\alpha(n)$	$E_n(n)$	$k^{\gamma(n)}$ by eq. (7)	$e^{g(n)}$ by eq. (13)	$e^{g(n)}$ by eq. (15)	$e^{g(n)}$ by eq. (17)	$\left(\frac{\sqrt{n}-1}{\sqrt{n}+\frac{\alpha(n)+\pi}{30}}\right)$ by eq. (19)
115	0.322158177	0.897116263	0.896647933254637	0.8970830973658035	0.8970840912397839	0.8971185968277765	0.8970909467245491
116	0.320896031	0.897564743	0.8970931224519181	0.8975275175006338	0.897527900478023	0.897562412562096	0.8975342342777326
117	0.324039846	0.897997289	0.8975326064110534	0.8979662316687815	0.8979660174806986	0.8980005322144554	0.8979598211213504
118	0.322094407	0.898431058	0.8979665059665459	0.898399360801867	0.8983985628956037	0.8984330765181445	0.8983938620438905
119	0.320030396	0.898855634	0.8983949383999705	0.8988270222751737	0.8988256538205951	0.8988601626549537	0.89882280816082
120	0.321449652	0.899271712	0.898818017573172	0.8992493300409609	0.8992474039367716	0.899281904388353	0.899237015246722
121	0.317625227	0.899681982	0.8992358540554108	0.8996663947557199	0.8996639236355971	0.8996984121906192	0.8996602400339961
122	0.324834413	0.900077197	0.8996485552447778	0.9000783239016961	0.9000753201402898	0.9001097933642321	0.9000486779115556
123	0.311363644	0.900499336	0.9000562254841823	0.9004852219029796	0.9004816976217807	0.9005161521578428	0.9004876976350763
124	0.316095229	0.900906604	0.9004589661721953	0.9008871902364495	0.9008831573095272	0.900917589877098	0.9008730267353279
125	0.311833214	0.901308578	0.9008568758690187	0.9012843275378378	0.9012797975974491	0.9013142049905908	0.9012776124462152
126	0.312771202	0.901696803	0.901250050397828	0.9016767297031673	0.9016717141452381	0.9017060932311836	0.9016636776612966
127	0.312018336	0.902079423	0.9016385829417293	0.9020644899857967	0.9020589999752776	0.9020933476929466	0.9020496586423263
128	0.310920299	0.902466729	0.9020225641365498	0.9024476990892992	0.9024417455653976	0.9024760589239289	0.9024320558706945
129	0.309668721	0.902843257	0.902402082159674	0.9028264452563821	0.9028200389376719	0.9028543150149761	0.9028104440802703
130	0.308633822	0.903215074	0.9027772228151212	0.9032008143540456	0.9031939657434588	0.9032282016847896	0.9031839358552052
131	0.305630293	0.903584309	0.9031480696150538	0.9035708899551691	0.9035636093448676	0.9035978023614151	0.9035583126048479
132	0.313429659	0.903930824	0.903514703857888	0.903936753416698	0.9039290508928317	0.9039631982603361	0.9039004410769711
133	0.299024683	0.904311065	0.9038772047031773	0.9042984839545996	0.9042903694019505	0.9043244684593384	0.9042960990843415
134	0.300440402	0.904665819	0.9042356492434216	0.9046561587157428	0.9046476418222585	0.9046816899703013	0.9046467103309881
135	0.30423526	0.905010643	0.9045901125729522	0.9050098528468519	0.9050009431080697	0.9050349378080645	0.9049873111650395
136	0.305177225	0.905357191	0.9049406678540313	0.9053596395606708	0.9053503462840362	0.9053842850565106	0.9053314400603566
137	0.306483292	0.905699238	0.9052873863802987	0.9057055901994743	0.905695922508555	0.9057298029319933	0.9056708780183221
138	0.295380826	0.90604982	0.905630337637691	0.9060477742960471	0.9060377411346451	0.9060715608442397	0.9060382208913295
139	0.291006903	0.906394809	0.9059695893629494	0.9063862596322524	0.9063758697684162	0.9064096264548414	0.9063846651748781
140	0.292739617	0.906732715	0.9063052075998308	0.9067211122952996	0.9067103743252397	0.9067440657334482	0.9067120132997448
141	0.296040514	0.907058533	0.9066372567531266	0.9070523967318177	0.9070413190837263	0.9070749430117704	0.9070319215030493
142	0.293910157	0.907386588	0.9069657996405891	0.907380175799836	0.9073687667376142	0.907402321035489	0.9073620845494251
143	0.29290743	0.907705986	0.9072908975428617	0.9077045108187651	0.9076927784456593	0.9077262610141693	0.9076859967538078
144	0.295892849	0.908021971	0.9076126102515025	0.9080254616174709	0.9080134138796202	0.908046822669269	0.9079965968387937
145	0.293927594	0.908340436	0.9079309961151864	0.908343086580524	0.9083307312704216	0.9083640642803239	0.9083162980987417
146	0.295042419	0.908649074	0.9082461120841667	0.9086574426927089	0.9086447874525793	0.9086780427293955	0.9086251017475645
147	0.287245831	0.908966854	0.9085580137530755	0.9089685855818677	0.908955637906961	0.9089888135438537	0.9089528181908137
148	0.290202958	0.909277393	0.9088667554021311	0.9092765695601519	0.9092633368019584	0.909296430937572	0.9092507649823885
149	0.285261335	0.909586047	0.9091723900368267	0.909581447663753	0.909567937033139	0.9096009478505989	0.9095651332846306
150	0.286313773	0.909884128	0.9094749694261632	0.9098832716911756	0.9098694902614431	0.909902415987378	0.9098617146245895
151	0.282099672	0.910185647	0.9097745441394904	0.9101820922401169	0.9101680469499894	0.9102008858535738	0.9101682357370078
152	0.28129088	0.910480228	0.9100711635820148	0.9104779587430134	0.9104636563995481	0.9104964067915658	0.9104634805526028

Continued on next page

Table S1 – *Continued from previous page*

n	$\alpha(n)$	$E_n(n)$	$k^{\gamma(n)}$ by eq. (7)	$e^{g(n)}$ by eq. (13)	$e^{g(n)}$ by eq. (15)	$e^{g(n)}$ by eq. (17)	$\left(\frac{\sqrt{n}-1}{\sqrt{n}+\frac{\alpha(n)+\pi}{30}}\right)$ by eq. (19)
153	0.286057028	0.910767888	0.9103648760290314	0.9107709195013085	0.9107563667827386	0.9107890270146666	0.9107422938462245
154	0.280611433	0.911061935	0.9106557286589333	0.9110610217184978	0.9110462251770056	0.9110787936401173	0.9110431199804202
155	0.279074201	0.911348569	0.9109437675850505	0.9113483115320017	0.9113332775964244	0.9113657527209138	0.9113316491560122
156	0.276138358	0.911636942	0.9112290378863659	0.911632834043914	0.9116175690223843	0.9116499492765083	0.9116208025045985
157	0.278348881	0.911915537	0.9115115836371555	0.9119146333506751	0.9118991434331978	0.9119314273224384	0.9118948602458131
158	0.279024447	0.912192773	0.9117914479355977	0.9121937525717105	0.9121780438326775	0.9122102298989216	0.9121699872040031
159	0.278715009	0.912465801	0.9120686729313925	0.9124702338770813	0.9124543122777248	0.9124863990984635	0.9124448785506651
160	0.278575447	0.912738657	0.9123432998524317	0.9127441185141816	0.9127279899049701	0.912759976092515	0.9127168019115725
161	0.275932151	0.913011438	0.9126153690305582	0.9130154468335271	0.9129991169565022	0.9130310011572198	0.9129921557841935
162	0.275985906	0.913279252	0.9128849199264508	0.9132842583136661	0.9132677328047244	0.913299513698287	0.9132586009086728
163	0.273586028	0.913546844	0.9131519911536701	0.913550591585251	0.9135338759763713	0.9135655522750256	0.9135284047079307
164	0.273233293	0.913810984	0.9134166205018971	0.9138144844543028	0.9137975841757195	0.9138291546235715	0.9137909458892004
165	0.273168267	0.914071952	0.9136788449593984	0.9140759739247	0.9140588943070259	0.9140903576793414	0.9140504372350308
166	0.272394218	0.91433075	0.9139387007347465	0.9143350962199226	0.9143178424962205	0.914349197598742	0.9143092569573825
167	0.272263955	0.914587789	0.9141962232778249	0.9145918868040805	0.9145744641118855	0.9146057097801645	0.9145642629861797
168	0.273224409	0.914839333	0.9144514473001464	0.914846380402253	0.9148287937855475	0.9148599288842917	0.914814459318102
169	0.271180307	0.915094461	0.9147044067945094	0.9150986110201664	0.9150808654313086	0.9151118888537442	0.915069425150726
170	0.271728006	0.915341251	0.914955135054019	0.9153486119632366	0.915330712264843	0.915361622932091	0.9153161550467114
171	0.270474484	0.915588108	0.9152036646904962	0.9155964158549967	0.9155783668217808	0.9156091636822483	0.9155648929981495
172	0.266903904	0.915834107	0.9154500276522982	0.9158420546549383	0.9158238609755062	0.9158545430042908	0.9158168280116508
173	0.265720172	0.916077065	0.9156942552415732	0.9160855596757834	0.9160672259543873	0.9160977921526953	0.9160611224570248
174	0.268189493	0.916315702	0.9159363781309686	0.9163269616002118	0.9163084923584627	0.9163389417530404	0.9162949427893312
175	0.266189188	0.916557147	0.9161764263798157	0.9165662904970624	0.9165476901756029	0.9165780218181815	0.9165369827755532
176	0.263570479	0.916797184	0.9164144294498072	0.9168035758370282	0.9167848487971666	0.9168150617639186	0.9167783964871219
177	0.266496332	0.917031348	0.9166504162201888	0.9170388465078638	0.9170199970331703	0.9170500904241792	0.9170051618050554
178	0.264324548	0.917264837	0.9168844150024809	0.9172721308291233	0.9172531631269893	0.9172831360657309	0.9172415856191454
179	0.261242212	0.917496619	0.917116453554748	0.9175034565664446	0.9174843747696069	0.9175142264024422	0.9174781167758899
180	0.262971527	0.917722218	0.917346559095433	0.9177328509453989	0.9177136591134272	0.9177433886091078	0.9177018292091842
181	0.261829167	0.917949223	0.9175747583167694	0.9179603406649173	0.9179410427856692	0.917970649334853	0.9179301591464217
182	0.264505777	0.918169944	0.9178010773977908	0.9181859519103135	0.9181665519013561	0.9181960347161335	0.9181480320216052
183	0.257376224	0.918401061	0.9180255420169471	0.9184097103659145	0.9183902120759124	0.9184195703893453	0.9183861134023451
184	0.260609816	0.918621305	0.9182481773643448	0.9186316412273129	0.9186120484373866	0.9186412815030552	0.9185991140293217
185	0.257874767	0.918844124	0.9184690081536239	0.9188517692132573	0.918832085638309	0.9188611927298711	0.9188237006813378
186	0.257243747	0.919059859	0.9186880586334821	0.919070118577188	0.9190503478671987	0.9190793282779578	0.9190418158051493
187	0.259146241	0.919269936	0.9189053525988622	0.9192867131184367	0.9192668588597335	0.9192957119022165	0.9192525626794241
188	0.257254496	0.91948974	0.9191209134018101	0.9195015761930957	0.9194816419095909	0.9195103669151363	0.9194700335382345
189	0.256050134	0.919703924	0.9193347639620189	0.9197147307245732	0.9196947198789754	0.9197233161973298	0.9196842783520585
190	0.253089893	0.919912927	0.9195469267770658	0.9199261992138411	0.9199061152088398	0.919934582207764	0.9199007211921831

Continued on next page

Table S1 – *Continued from previous page*

**n**	$\alpha(n)$	$E_n(n)$	$k^{\gamma(n)}$ by eq. (7)	$e^{g(n)}$ by eq. (13)	$e^{g(n)}$ by eq. (15)	$e^{g(n)}$ by eq. (17)	$\left(\frac{\sqrt{n}-1}{\sqrt{n}+\frac{\alpha(n)+\pi}{30}}\right)$ by eq. (19)
191	0.247742035	0.920120184	0.9197574239323567	0.9201360037493894	0.9201158499288125	0.9201441869936964	0.9201207470930258
192	0.258636278	0.920320008	0.9199662771107833	0.9203441660168944	0.9203239456668401	0.9203521522003264	0.9203034292231436
193	0.246864902	0.920538256	0.9201735076021073	0.9205507073086114	0.920530423658556	0.9205584990801718	0.9205342814023018
194	0.250462253	0.920746952	0.9203791363120771	0.9207556485325024	0.9207353047563822	0.9207632485021794	0.9207298420102844
195	0.250891657	0.920946183	0.9205831837712884	0.920959010221104	0.9209386094383748	0.9209664209605782	0.9209307890507693
196	0.248726209	0.921150591	0.9207856701437956	0.9211608125401496	0.9211403578168216	0.9211680365834852	0.9211358470541067
197	0.246905623	0.921350211	0.9209866152354829	0.9213610752969476	0.9213405696465986	0.9213681151412695	0.9213386155172871
198	0.248489767	0.921551994	0.9211860385022029	0.9215598179485295	0.9215392643332958	0.9215666760546849	0.9215324923596074
199	0.247016905	0.921748567	0.9213839590576911	0.9217570596095713	0.9217364609411168	0.9217637384027781	0.921731508859502
200	0.248216604	0.921942136	0.9215803956812614	0.9219528190600981	0.9219321782005624	0.9219593209305782	0.9219232866164498
201	0.241380385	0.922135157	0.921775366825292	0.9221471147529764	0.9221264345159028	0.9221534420565766	0.9221309160707892
202	0.247647145	0.922321572	0.9219688906225073	0.9223399648212044	0.9223192479724471	0.9223461198800033	0.9223089323364336
203	0.243942046	0.922516013	0.9221609848930626	0.9225313870850028	0.9225106363436157	0.9225373721879054	0.9225069522418567
204	0.249421341	0.92269996	0.9223516671514377	0.9227213990587164	0.9227006170978216	0.9227272164620355	0.922683922353511
205	0.24277621	0.922896807	0.9225409546131452	0.9229100179575285	0.9228892074051671	0.9229156698855544	0.9228854155053539
206	0.243257581	0.923082093	0.9227288642012611	0.9230972607039977	0.9230764241439621	0.9231027493495556	0.9230703291545611
207	0.243604046	0.923272744	0.9229154125527801	0.9232831439344199	0.9232622839070688	0.9232884714594162	0.9232541916145484
208	0.241746125	0.923456142	0.923100616024804	0.9234676840050233	0.9234468030080785	0.9234728525409795	0.9234413996490152
209	0.241663353	0.923639783	0.9232844907005673	0.9236508969979986	0.9236299974873263	0.9236559086465754	0.9236235292817622
210	0.240946001	0.923820935	0.9234670523953041	0.9238327987273732	0.9238118831177476	0.9238376555608825	0.9238057016588577
211	0.235943836	0.924001734	0.9236483166619637	0.9240134047447309	0.9239924754105824	0.9240181088066381	0.9239956036005131
212	0.246401574	0.924174666	0.9238282987967756	0.9241927303447837	0.9241717896209312	0.9241972836501994	0.9241517384145788
213	0.237914986	0.924358572	0.9240070138446739	0.9243707905707995	0.9243498407531681	0.9243751951069614	0.9243464057495587
214	0.240345448	0.924530981	0.9241844766045789	0.9245476002198916	0.9245266435662135	0.9245518579466356	0.924516940178145
215	0.236721238	0.92470952	0.9243607016345464	0.9247231738481702	0.9247022125786736	0.9247272866983935	0.9246989063441731
216	0.239728914	0.924879817	0.9245357032567848	0.9248975257757652	0.924876562073847	0.9249014956558788	0.9248658286415369
217	0.242631891	0.925050695	0.9247094955625443	0.9250706700917213	0.9250497061046064	0.9250744988820934	0.9250318024058231
218	0.236670114	0.925227841	0.9248820924168845	0.925242620658768	0.9252216584981551	0.925246310214159	0.925214998633884
219	0.235276651	0.925397113	0.9250535074633206	0.9254133911179714	0.9253924328606663	0.9254169432679606	0.9253875276910448
220	0.237404583	0.925564281	0.9252237541283546	0.9255829948932695	0.9255620425818041	0.925586411442672	0.9255516231695312
221	0.234993525	0.925733771	0.9253928456258932	0.925751445195894	0.9257305008391333	0.9257547279251699	0.9257239483812896
222	0.235656297	0.92589991	0.9255607949615565	0.925918755028683	0.92589782060242	0.925921905694339	0.9258888073884812
223	0.235422581	0.926065912	0.9257276149368805	0.9260849371902873	0.9260640146378253	0.9260879575252693	0.9260543974903449
224	0.237407189	0.926229467	0.9258933181534158	0.926250004279273	0.9262290955119966	0.9262528959933521	0.9262143423524015
225	0.230620811	0.92639749	0.926057917016727	0.9264139686981243	0.9263930755960588	0.9264167334782748	0.926391136303733
226	0.233485763	0.926560855	0.9262214237402945	0.9265768426571492	0.9265559670695079	0.9265794821679184	0.9265471161703052
227	0.233104595	0.92672386	0.9263838503493215	0.9267386381782894	0.9267177819240116	0.9267411540621613	0.9267086604728059
228	0.231714375	0.926884387	0.9265452086844491	0.926899367098839	0.9268785319671156	0.9269017609765906	0.9268711973999758

Continued on next page

Table S1 – *Continued from previous page*

**n**	$\alpha(n)$	$E_n(n)$	$k^{\gamma(n)}$ by eq. (7)	$e^{g(n)}$ by eq. (13)	$e^{g(n)}$ by eq. (15)	$e^{g(n)}$ by eq. (17)	$\left(\frac{\sqrt{n}-1}{\sqrt{n}+\frac{\alpha(n)+\pi}{30}}\right)$ by eq. (19)
229	0.229464507	0.927045606	0.9267055104053832	0.9270590410750746	0.9270382288258641	0.927061314546125	0.9270344233634832
230	0.231597258	0.927201715	0.926864766994433	0.9272176715857973	0.9271968839503308	0.9272198262285497	0.9271877347028546
231	0.229769788	0.927358276	0.9270229897599669	0.9273752699357903	0.9273545086170684	0.9273773073079694	0.9273480387873639
232	0.231437664	0.927513835	0.9271801898397856	0.9275318472591966	0.9275111139324732	0.9275337688981766	0.9275002767446666
233	0.232626177	0.927667019	0.9273363782044155	0.927687414522814	0.927666710836074	0.9276892219459429	0.9276524940028126
234	0.229248474	0.92782277	0.9274915656603253	0.9278419825293155	0.9278213101037405	0.9278436772342312	0.9278128977408674
235	0.228146073	0.927976936	0.9276457628530669	0.9279955619203916	0.927974922350818	0.9279971453853337	0.9279677405412771
236	0.2297601	0.928127713	0.927798980270343	0.9281481631798217	0.9281275580351893	0.9281496368639363	0.9281161775463534
237	0.227372445	0.92828311	0.9279512282450042	0.9282997966364732	0.9282792274602647	0.928301161980112	0.9282716555436168
238	0.226583338	0.928434646	0.9281025169579757	0.9284504724672306	0.9284299407779035	0.9284517308922452	0.9284229853636314
239	0.224467944	0.928585778	0.9282528564411184	0.9286002006998584	0.9285797079912678	0.9286013536098883	0.9285760090725481
240	0.226385629	0.928732924	0.9284022565800221	0.9287489912157983	0.9287285389576112	0.9287500399965536	0.9287200887784801
241	0.224407579	0.928881744	0.9285507271167371	0.9288968537529014	0.9288764433910041	0.9288977997724406	0.9288709800828993
242	0.224994433	0.929028047	0.928698277652443	0.9290437979080997	0.9290234308649963	0.9290446425171017	0.9290158796953657
243	0.224105755	0.92917297	0.9288449176500561	0.9291898331400156	0.92916951081522	0.9291905776720478	0.9291627961753531
244	0.225767968	0.929316888	0.9289906564367798	0.9293349687715147	0.9293146925419336	0.9293356145432949	0.929303794420673
245	0.222423288	0.929464081	0.9291355032065967	0.9294792139921989	0.9294589852125088	0.9294797623038531	0.9294537647696762
246	0.22381442	0.929607094	0.9292794670227046	0.9296225778608458	0.9296023978638621	0.9296230299961609	0.9295935497103311
247	0.221598953	0.929749144	0.9294225568198995	0.9297650693077921	0.9297449394048317	0.9297654265344644	0.9297395440955446
248	0.222122837	0.929892433	0.9295647814069048	0.9299066971372654	0.929886618618502	0.9299069607071443	0.9298793151955039
249	0.221830586	0.930032545	0.9297061494686495	0.9300474700296637	0.9300274441644765	0.9300476411789912	0.930019837103775
250	0.219608502	0.930172086	0.9298466695684957	0.9301873965437856	0.9301674245811017	0.9301874764934307	0.9301632779813643
251	0.218062227	0.930310118	0.9299863501504184	0.9303264851190102	0.9303065682876401	0.9303264750747002	0.9303045612464006
252	0.220914655	0.930446046	0.9301251995411366	0.9304647440774308	0.9304448835863985	0.9304646452299781	0.9304364797903026
253	0.219761685	0.930585304	0.9302632259521986	0.9306021816259421	0.9305823786648076	0.9306019951514659	0.9305753630347394
254	0.215331343	0.93072177	0.9304004374820221	0.9307388058582807	0.9307190615974582	0.9307385329184263	0.9307197694413647
255	0.219303688	0.930855033	0.9305368421178906	0.9308746247570235	0.9308549403480922	0.9308742664991768	0.9308471354625929
256	0.219426067	0.93099022	0.9306724477379061	0.9310096461955425	0.9309900227715525	0.9310092037530405	0.930981156054137
257	0.221168271	0.931125117	0.930807262112901	0.9311438779399166	0.9311243166156891	0.931143352432255	0.9311112824768711
258	0.218061124	0.931260549	0.9309412929083085	0.9312773276508046	0.931257829523226	0.9312767201838409	0.9312499544099633
259	0.216598865	0.931394651	0.9310745476859938	0.931410002885277	0.9313905690335885	0.9314093145514306	0.9313846881668785
260	0.218864221	0.931525982	0.931207033906046	0.9315419110986097	0.9315225425846906	0.931541142977058	0.9315115249719319
261	0.216412999	0.931658161	0.9313387589285328	0.9316730596460394	0.9316537575146862	0.9316722128029112	0.931646629962743
262	0.217178896	0.931788702	0.9314697300152183	0.9318034557844826	0.9317842210636822	0.9318025312730472	0.9317748437655667
263	0.21640527	0.931917908	0.9315999543312443	0.9319331066742188	0.931913940375417	0.9319321055350723	0.9319052544481223
264	0.217604802	0.932045114	0.9317294389467776	0.9320620193805376	0.932042922498903	0.9320609426417863	0.9320311829981183
265	0.215334095	0.932175504	0.9318581908386219	0.9321902008753529	0.9321711743900359	0.9321890495527929	0.9321629749341526
266	0.215019838	0.932302098	0.9319862168917974	0.9323176580387827	0.9322987029131699	0.9323164331360768	0.9322903332077842

Continued on next page

Table S1 – *Continued from previous page*

n	$\alpha(n)$	$E_n(n)$	$k^{\gamma(n)}$ by eq. (7)	$e^{g(n)}$ by eq. (13)	$e^{g(n)}$ by eq. (15)	$e^{g(n)}$ by eq. (17)	$\left(\frac{\sqrt{n}-1}{\sqrt{n}+\frac{\alpha(n)+\pi}{30}}\right)$ by eq. (19)
267	0.214972314	0.932428904	0.9321135239010875	0.9324443976606978	0.9324255148426606	0.9324431001695488	0.9324164759317541
268	0.214020024	0.932553867	0.9322401185725534	0.9325704264422358	0.932551616864377	0.932569057342558	0.9325436216727405
269	0.214343267	0.932679226	0.9323660075250185	0.932695750997288	0.9326770155771821	0.9326943112573747	0.9326676633600509
270	0.209333377	0.932802428	0.9324911972915216	0.9328203778539528	0.9328017174943822	0.932818868430641	0.9328010406514218
271	0.207533595	0.932924163	0.9326156943207418	0.9329443134559615	0.9329257290451493	0.9329427352947943	0.9329276791656449
272	0.215279596	0.933044801	0.9327395049783932	0.9330675641640742	0.9330490565759116	0.9330659181994605	0.9330357485742301
273	0.213494845	0.933173875	0.9328626355485932	0.9331901362574481	0.9331717063517196	0.9331884234128189	0.9331610178296268
274	0.212869745	0.933295153	0.9329850922352011	0.9333120359349782	0.9332936845575811	0.9333102571229412	0.933283445913472
275	0.211193046	0.933418686	0.9331068811631326	0.9334332693166115	0.9334149972997721	0.933431425439102	0.9334071708270355
276	0.209776487	0.933538997	0.9332280083796455	0.933553842444634	0.9335356506071204	0.9335519343930643	0.93352974819327
277	0.210424886	0.933658481	0.9333484798556021	0.9336737612849334	0.9336556504322644	0.9336717899403394	0.933647834020438
278	0.20948923	0.933777674	0.933468301486705	0.9337930317282362	0.933775002652886	0.9337909979614212	0.9337682198853623
279	0.210112361	0.933894951	0.9335874790947092	0.9339116595913204	0.9338937130729202	0.9339095642629964	0.9338850785782066
280	0.205483174	0.934011979	0.9337060184286107	0.9340296506182039	0.9340117874237404	0.934027494579132	0.9340110187076294
281	0.204032667	0.934127116	0.9338239251658111	0.9341470104813111	0.9341292313653202	0.9341447945724376	0.9341304409838895
282	0.212342403	0.934241095	0.9339412049132606	0.9342637447826151	0.9342460504873742	0.9342614698352066	0.9342312554399065
283	0.207262032	0.934362415	0.9340578632085768	0.9343798590547587	0.9343622503104737	0.9343775258905348	0.9343561333158801
284	0.207555387	0.934479513	0.9341739055211444	0.9344953587621535	0.9344778362871438	0.9344929681934165	0.9344705055993635
285	0.203744886	0.934595	0.934289337253192	0.9346102493020572	0.9345928138029372	0.9346078021318215	0.9345918002410818
286	0.208236173	0.934707646	0.934404163740848	0.934724536005631	0.9347071881774888	0.9347220330277484	0.9346972804377888
287	0.207218307	0.934820972	0.9345183902551777	0.9348382241389764	0.9348209646655484	0.9348356661382605	0.9348122618598038
288	0.206661219	0.934933962	0.9346320220031992	0.9349513189041523	0.9349341484579962	0.9349487066565008	0.9349258095650885
289	0.20600681	0.935047454	0.9347450641288808	0.9350638254401722	0.9350467446828359	0.9350611597126864	0.9350389492452374
290	0.20268556	0.935158384	0.9348575217141193	0.9351757488239829	0.9351587584061716	0.9351730303750871	0.9351563579132579
291	0.206306166	0.935267931	0.9349693997796992	0.9352870940714255	0.9352701946331646	0.9352843236509821	0.9352605711999202
292	0.206948441	0.935377369	0.9350807032862347	0.9353978661381764	0.9353810583089733	0.9353950444876014	0.9353696372788197
293	0.206028893	0.93548787	0.9351914371350943	0.9355080699206726	0.9354913543196742	0.9355051977730473	0.9354809714309091
294	0.203361834	0.935600035	0.9353016061693061	0.9356177102570181	0.9356010874931671	0.9356147883372008	0.9355949006288764
295	0.201718469	0.935711387	0.9354112151744488	0.9357267919278749	0.9357102626000617	0.9357238209526094	0.9357064148571927
296	0.203283359	0.935820259	0.9355202688795244	0.9358353196573359	0.9358188843545499	0.935832300335359	0.9358115921790126
297	0.201796731	0.935929515	0.9356287719578156	0.9359432981137829	0.9359269574152598	0.9359402311459302	0.9359217232542487
298	0.20016585	0.936037133	0.9357367290277265	0.9360507319107275	0.9360344863860954	0.9360476179900376	0.936031566054547
299	0.201204667	0.93614347	0.9358441446536093	0.9361576256076387	0.9361414758170601	0.9361544654194549	0.9361360783514886
300	0.199435675	0.936250314	0.9359510233465742	0.9362639837107523	0.9362479302050654	0.9362607779328241	0.9362450931310481
301	0.201014148	0.936355153	0.9360573695652855	0.9363698106738679	0.9363538539947247	0.93636655997645	0.9363475887850484
302	0.200037348	0.936460983	0.9361631877167432	0.9364751108991308	0.9364592515791331	0.9364718159450804	0.9364541324065615
303	0.197982344	0.936564584	0.9362684821570496	0.9365798887377988	0.9365641273006319	0.9365765501826722	0.9365620750923098
304	0.201364756	0.9366671	0.9363732571921634	0.9366841484909963	0.9366684854515606	0.9366807669831434	0.9366598187780402

Continued on next page

Table S1 – *Continued from previous page*

**n**	$\alpha(n)$	$E_n(n)$	$k^{\gamma(n)}$ by eq. (7)	$e^{g(n)}$ by eq. (13)	$e^{g(n)}$ by eq. (15)	$e^{g(n)}$ by eq. (17)	$\left(\frac{\sqrt{n}-1}{\sqrt{n}+\frac{\alpha(n)+\pi}{30}}\right)$ by eq. (19)
305	0.200461218	0.93677077	0.9364775170786386	0.9367878944104542	0.9367723302749946	0.9367844705911115	0.9367646867699504
306	0.202949569	0.936874061	0.9365812660243517	0.9368911306992365	0.9368756659654697	0.9368876652026191	0.9368630284406266
307	0.199686664	0.936976471	0.9366845081892144	0.9369938615124541	0.9369784966696936	0.9369903549658462	0.9369710700255418
308	0.201196736	0.937077354	0.9367872476858748	0.9370960909579659	0.9370808264872452	0.9370925439818091	0.9370701564255438
309	0.196298158	0.937182566	0.9368894885804055	0.9371978230970665	0.9371826594712609	0.9371942363050482	0.9371800758875805
310	0.197684788	0.937284049	0.9369912348929793	0.9372990619451633	0.9372839996291081	0.9372954359443026	0.9372783983378558
311	0.198575385	0.937384893	0.9370924905985334	0.9373998114724407	0.9373848509230491	0.9373961468631734	0.9373771170732573
312	0.197211516	0.937485902	0.9371932596274222	0.9375000756045121	0.9374852172708902	0.937496372980774	0.9374793234413713
313	0.197229216	0.937586227	0.9372935458660574	0.9375998582230611	0.9375851025466219	0.9375961181723714	0.9375786207109454
314	0.197554003	0.937684688	0.9373933531575382	0.9376991631664723	0.9376845105810465	0.9376953862700134	0.937676906888271
315	0.197874483	0.937784342	0.9374926853022698	0.9377979942304485	0.9377834451623954	0.9377941810631472	0.9377747331237863
316	0.195527565	0.937882319	0.9375915460585712	0.9378963551686202	0.9378819100369354	0.9378925062992258	0.937876757389018
317	0.199374732	0.937979487	0.9376899391432728	0.9379942496931429	0.9379799089095644	0.9379903656843047	0.9379674982337601
318	0.195806056	0.938077133	0.9377878682323032	0.9380916814752833	0.9380774454443968	0.9380877628836275	0.9380707233663934
319	0.192966728	0.938175013	0.9378853369612661	0.9381886541459977	0.9381745232653389	0.9381847015222021	0.9381722067211101
320	0.19500135	0.938270878	0.9379823489260073	0.9382851712964982	0.938271145956655	0.9382811851853667	0.9382647559394796
321	0.191947297	0.938367464	0.9380789076831723	0.9383812364788107	0.9383673170635223	0.9383772174193463	0.9383656969674176
322	0.195527879	0.938462293	0.9381750167507534	0.9384768532063222	0.9384630400925779	0.938472801731799	0.938454683875249
323	0.191196351	0.938557838	0.9382706796086289	0.9385720249543202	0.9385583185124556	0.9385679415923542	0.9385569363704233
324	0.196493314	0.938651216	0.9383658996990917	0.9386667551605212	0.9386531557543137	0.9386626404331403	0.9386420962774576
325	0.19421148	0.938747014	0.9384606804273702	0.9387610472255918	0.9387475552123542	0.9387569016493046	0.9387399226303473
326	0.19103435	0.938840156	0.9385550251621393	0.9388549045136605	0.9388415202443325	0.9388507285995238	0.938838849491076
327	0.194100372	0.938932776	0.9386489372360237	0.9389483303528197	0.93893505417206	0.9389441246065061	0.9389265941037898
328	0.19375872	0.939025748	0.9387424199460928	0.9390413280356217	0.9390281602818962	0.939037092957485	0.939019783222213
329	0.192009944	0.939119299	0.9388354765543458	0.9391339008195644	0.9391208418252344	0.9391296369047044	0.9391149635493007
330	0.192228244	0.939211228	0.9389281102881912	0.9392260519275692	0.9392131020189782	0.9392217596658962	0.9392063483843275
331	0.193180818	0.939302771	0.9390203243409161	0.9393177845484522	0.939304944046011	0.9393134644247492	0.9392960638398832
332	0.191762812	0.939394508	0.9391121218721489	0.9394091018373857	0.9393963710556562	0.9394047543313716	0.9393894217083635
333	0.193483001	0.939484622	0.939203506008314	0.939500006916354	0.9394873861641321	0.9394956325027439	0.9394770116654949
334	0.192998132	0.93957704	0.9392944798430796	0.9395905028746002	0.9395779924549967	0.9395861020231665	0.9395679605443044
335	0.189820411	0.939667032	0.9393850464377967	0.9396805927690662	0.9396681929795873	0.9396761659446977	0.9396630854060445
336	0.188962324	0.939757855	0.9394752088219328	0.9397702796248258	0.9397579907574517	0.9397658272875866	0.9397538558956965
337	0.186831464	0.939847226	0.9395649699934969	0.9398595664355106	0.9398473887767732	0.9398550890406976	0.9398463851314021
338	0.18767431	0.939935716	0.9396543329194587	0.939948456163728	0.9399363899947876	0.9399439541619284	0.9399334737574861
339	0.190143765	0.940023668	0.9397433005361603	0.9400369517414744	0.9400249973381946	0.9400324255786214	0.9400174243052167
340	0.190308726	0.940112361	0.9398318757497217	0.9401250560705395	0.9401132137035616	0.940120506187968	0.9401048917703659
341	0.18886772	0.940200254	0.9399200614364394	0.940212772022906	0.9402010419577218	0.9402081988574071	0.9401946845374887
342	0.190219147	0.940286701	0.940007860443179	0.940300102441141	0.9402884849381652	0.9402955064250162	0.9402793843418699

Continued on next page

Table S1 – *Continued from previous page*

n	$\alpha(n)$	$E_n(n)$	$k^{\gamma(n)}$ by eq. (7)	$e^{g(n)}$ by eq. (13)	$e^{g(n)}$ by eq. (15)	$e^{g(n)}$ by eq. (17)	$\left(\frac{\sqrt{n}-1}{\sqrt{n}+\frac{\alpha(n)+\pi}{30}}\right)$ by eq. (19)
343	0.185755333	0.940373604	0.9400952755877608	0.9403870501387834	0.9403755454534238	0.9403824316998971	0.940373494889323
344	0.190465596	0.94045921	0.9401823096593405	0.9404736179007227	0.9404622262834503	0.9404689774625549	0.940451794917504
345	0.1885258	0.940546578	0.940268965418782	0.9405598084835731	0.940548530179991	0.9405551464652712	0.9405409006467943
346	0.188695807	0.940632273	0.9403552455990264	0.9406456246160423	0.9406344598669526	0.9406409414324715	0.9406260914229739
347	0.186024944	0.940717663	0.9404411529054529	0.9407310689992923	0.9407200180407636	0.9407263650610863	0.9407156684908397
348	0.187119179	0.940802491	0.9405266900162365	0.9408161443072968	0.9408052073707295	0.9408114200209072	0.9407985759570545
349	0.187253734	0.940888294	0.9406118595826971	0.9409008531871912	0.9408900304993821	0.9408961089549361	0.9408827268963479
350	0.187237041	0.940973002	0.9406966642296465	0.9409851982596186	0.9409744900428247	0.9409804344797309	0.9409667700683618
351	0.185017479	0.941057706	0.9407811065557267	0.941069182119069	0.9410585885910703	0.9410643991857439	0.941054121882529
352	0.184833044	0.941141278	0.9408651891337454	0.941152807334214	0.9411423287083751	0.9411480056376558	0.9411377256444566
353	0.183122624	0.941224559	0.9409489145110046	0.9412360764482364	0.9412257129335676	0.9412312563747045	0.9412235090317853
354	0.182470568	0.941306968	0.9410322852096255	0.9413189919791537	0.9413087437803711	0.9413141539110087	0.9413071799555622
355	0.182477927	0.94138901	0.9411153037268668	0.9414015564201383	0.9413914237377224	0.9413967007358861	0.941389408374737
356	0.185697913	0.941470549	0.9411979725354398	0.9414837722398306	0.9414737552700853	0.9414788993141675	0.9414659791952212
357	0.187240841	0.941551883	0.941280294083817	0.9415656418826491	0.9415557408177584	0.9415607520865051	0.9415449904150723
358	0.185829863	0.941634639	0.9413622707965373	0.941647167769095	0.9416373827971799	0.9416422614696771	0.9416285392645571
359	0.181850639	0.941717695	0.9414439050745058	0.9417283522960517	0.9417186836012257	0.9417234298568864	0.9417159739249982
360	0.181506533	0.941798645	0.941525199295289	0.9418091978370806	0.9417996455995049	0.9418042596180558	0.9417970760711116
361	0.183883562	0.941878992	0.9416061558134058	0.9418897067427121	0.9418802711386492	0.9418847531001187	0.9418733728081214
362	0.18053664	0.941959218	0.941686776960614	0.9419698813407316	0.9419605625425991	0.9419649126273039	0.9419587397126287
363	0.18028192	0.942038568	0.9417670650461927	0.9420497239364616	0.9420405221128844	0.9420447405014183	0.9420386965752872
364	0.180035217	0.942117678	0.9418470223572194	0.9421292368130403	0.9421201521289022	0.9421242390021236	0.9421183125105198
365	0.180232041	0.942196454	0.9419266511588444	0.9422084222316942	0.9421994548481897	0.9422034103872097	0.942196877999894
366	0.180419684	0.942274865	0.9420059536945603	0.9422872824320088	0.9422784325066922	0.9422822568928633	0.9422751370077447
367	0.184386377	0.942352841	0.942084932186467	0.9423658196321927	0.9423570873190287	0.9423607807339335	0.942346915911752
368	0.178777083	0.942432143	0.9421635888355336	0.9424440360293408	0.9424354214787524	0.9424389841041924	0.9424339847347905
369	0.178707684	0.942509482	0.9422419258218564	0.9425219337996903	0.9425134371586077	0.9425168691765923	0.9425117060786605
370	0.178273588	0.942586518	0.9423199453049119	0.9425995150988759	0.9425911365107833	0.9425944381035191	0.9425897057947634
371	0.177372551	0.942663232	0.9423976494238078	0.9426767820621786	0.9426685216671618	0.942671693017042	0.9426681496026333
372	0.183799663	0.942739437	0.9424750402975286	0.9427537368047733	0.9427455947395643	0.942748636029159	0.9427344087478766
373	0.181149071	0.942820282	0.9425521200251786	0.9428303814219704	0.9428223578199943	0.9428252692320395	0.9428150739652064
374	0.177814954	0.94289699	0.9426288906862216	0.9429067179894555	0.9428988129808747	0.9429015946982626	0.942896527683076
375	0.180884282	0.942972863	0.9427053543407153	0.9429827485635252	0.9429749622752831	0.9429776144810529	0.9429673299315758
376	0.177752655	0.943048976	0.9427815130295452	0.9430584751813185	0.9430508077371836	0.9430533306145112	0.9430478387021806
377	0.177130333	0.94312445	0.9428573687746516	0.9431338998610467	0.9431263513816551	0.9431287451138443	0.9431239958550536
378	0.177229398	0.943200666	0.9429329235792568	0.9432090246022178	0.9432015952051159	0.9432038599755884	0.9431986932700889
379	0.176813126	0.943275363	0.943008179428086	0.9432838513858596	0.943276541185545	0.9432786771778323	0.9432739233447984
380	0.179437937	0.943349605	0.9430831382875872	0.9433583821747378	0.9433511912827013	0.943353198680435	0.9433439805187542

Continued on next page

Table S1 – *Continued from previous page*

n	$\alpha(n)$	$E_n(n)$	$k^{\gamma(n)}$ by eq. (7)	$e^{g(n)}$ by eq. (13)	$e^{g(n)}$ by eq. (15)	$e^{g(n)}$ by eq. (17)	$\left(\frac{\sqrt{n}-1}{\sqrt{n}+\frac{\alpha(n)+\pi}{30}}\right)$ by eq. (19)
381	0.175912723	0.943423588	0.9431578021061463	0.9434326189135726	0.9434255474383378	0.9434274264252416	0.9434236089768803
382	0.175668815	0.943496983	0.9432321728143006	0.9435065635292508	0.9434996115764148	0.9435013623362952	0.9434976837380271
383	0.175735764	0.943570983	0.9433062523249479	0.9435802179310359	0.9435733856033083	0.9435750083200468	0.9435709730726694
384	0.175640187	0.943644067	0.9433800425335538	0.9436535840107745	0.9436468714080158	0.9436483662655606	0.9436442362673193
385	0.175048709	0.943716911	0.9434535453183543	0.9437266636430999	0.9437200708623608	0.9437214380447181	0.9437180057022317
386	0.17485158	0.943789392	0.9435267625405581	0.9437994586856332	0.9437929858211913	0.9437942255124182	0.9437908627997479
387	0.175264501	0.943861595	0.9435996960445434	0.943871970979181	0.9438656181225785	0.9438667305067747	0.943862468622944
388	0.174381369	0.943933613	0.9436723476580536	0.9439442023479304	0.9439379695880109	0.9439389548493113	0.9439358562433972
389	0.174137508	0.94400531	0.9437447191923897	0.9440161545996419	0.9440100420225861	0.9440109003451532	0.9440079495349613
390	0.178406146	0.94407673	0.9438168124425999	0.9440878295258385	0.9440818372152	0.9440825687832167	0.9440726163036653
391	0.172888884	0.944148846	0.9438886291876665	0.9441592289019928	0.9441533569387337	0.9441539619363957	0.9441525145665134
392	0.179383874	0.944219314	0.9439601711906905	0.9442303544877114	0.9442246029502367	0.9442250815617459	0.9442131309775688
393	0.173629894	0.944292823	0.9440314401990736	0.944301208026917	0.9442955769911086	0.9442959294006658	0.9442928427617877
394	0.173475172	0.944363335	0.9441024379446968	0.9443717912480271	0.9443662807872777	0.9443665071790761	0.944363434404166
395	0.17296633	0.944433629	0.9441731661440981	0.944442105864131	0.9444367160493776	0.9444368166075958	0.9444343169612237
396	0.173421765	0.944503426	0.944243626498646	0.9445121535731644	0.9445068844729204	0.9445068593817162	0.9445034158116833
397	0.173322026	0.944572968	0.944313820694712	0.9445819360580805	0.944576787738469	0.9445766371819725	0.9445731258320459
398	0.172689215	0.944642605	0.94438375040384	0.9446514549870201	0.944646427511805	0.9446461516741129	0.9446434108890647
399	0.172954924	0.944711789	0.9444534172829133	0.9447207120134787	0.9447158054440968	0.9447154045092656	0.9447120251926467
400	0.175720929	0.944780606	0.9445228229743197	0.9447897087764713	0.9447849231720628	0.9447843973241031	0.9447764669892423
402	0.176268705	0.944917111	0.9446608572921787	0.9449269279966885	0.9449223844906146	0.9449216093682163	0.9449124000478768
410	0.169300782	0.94545798	0.9452028658467642	0.9454657124644096	0.945462139278607	0.9454603744057201	0.945460339895549
420	0.168246138	0.946111084	0.9458584949371824	0.9461173911895865	0.9461150340253028	0.946112048437151	0.9461116485734045
430	0.165730138	0.946740062	0.94649113698393	0.9467461685566142	0.9467450291716224	0.9467408410482332	0.9467424163947488
432	0.166781025	0.946863315	0.9466150243364758	0.9468692929894853	0.9468683972255154	0.946863970762408	0.9468636022650105
440	0.164883802	0.947348446	0.9471021046561786	0.9473533542388675	0.9473534327768489	0.9473480601291427	0.94734917963445
448	0.162696755	0.947819146	0.9475760895911032	0.947824371189761	0.9478254232147266	0.9478191157811395	0.9478221953082772
450	0.162116265	0.947935223	0.947692608018134	0.9479401554369473	0.9479414506298299	0.9479349112675303	0.9479385357091623
460	0.162269748	0.948504377	0.9482637646157531	0.9485076869596183	0.9485101963050812	0.9485025078108396	0.9485043988423635
468	0.159119955	0.948945063	0.9487074576270792	0.9489485336528113	0.9489520117893153	0.9489434164831443	0.94894877271232
470	0.15810116	0.949052841	0.9488166083773932	0.9490569801187324	0.9490607000361778	0.9490518797449344	0.949058438242574
480	0.156109588	0.9495861	0.9493520974897725	0.9495889905995718	0.949593916567384	0.949583981550142	0.9495920707689401
482	0.156321785	0.94969092	0.9494571921620887	0.9496933979697483	0.9496985645309127	0.9496884085928474	0.9496959342454288
490	0.154414058	0.950102812	0.9498711213858011	0.9501046054432137	0.9501107321197196	0.9500996991710391	0.9501089479383394
492	0.155225322	0.950204023	0.9499730241419331	0.9502058345352872	0.9502122006480257	0.9502009501033867	0.9502088027297639
500	0.153881704	0.9506037	0.9503745069602687	0.9506046492569294	0.9506119705871324	0.9505998562157595	0.9506086772949623
510	0.151477682	0.951090479	0.9508630241122356	0.9510898897514838	0.9510983990613584	0.9510852194834952	0.9510963061206105
520	0.150627134	0.951561716	0.9513373906986604	0.9515610426898794	0.9515707327697649	0.9515565039045616	0.9515677304531903

Continued on next page

Table S1 – *Continued from previous page*

**n**	$\alpha(n)$	$E_n(n)$	$k^{\gamma(n)}$ by eq. (7)	$e^{g(n)}$ by eq. (13)	$e^{g(n)}$ by eq. (15)	$e^{g(n)}$ by eq. (17)	$\left(\frac{\sqrt{n}-1}{\sqrt{n}+\frac{\alpha(n)+\pi}{30}}\right)$ by eq. (19)
522	0.150138624	0.951654574	0.9514306257218308	0.9516536423666656	0.9516635676935535	0.9516491308548352	0.9516608326953999
530	0.148848244	0.952020376	0.9517982769717799	0.9520187763200675	0.9520296394984924	0.952014376965406	0.9520270769385083
540	0.147739112	0.952466353	0.9522463095626434	0.9524637153540275	0.9524757435631217	0.9524594626808419	0.9524727581678153
550	0.146278151	0.952899787	0.9526820750646597	0.9528964445470745	0.95290962938057	0.9528923451672542	0.9529067556792429
560	0.144682059	0.953322062	0.9531061232637709	0.953317511924003	0.9533318446886985	0.9533135718632993	0.9533293114924702
570	0.143987835	0.953731625	0.9535189700557113	0.9537274316925213	0.9537429034539708	0.9537236564384715	0.9537395571231045
572	0.143638388	0.953813263	0.9536002384162898	0.9538081209606519	0.9538238194309988	0.9538043792821868	0.9538205962906491
582	0.141976255	0.954211548	0.9540002806720663	0.9542052986209262	0.9542221251050612	0.9542017276847743	0.9542194265926441
612	0.138461532	0.955347277	0.9551410406159037	0.9553377608767718	0.9553579149515807	0.95533472725983	0.95535509786387
632	0.136341424	0.956058034	0.9558560212057117	0.9560474423703661	0.9560697668600183	0.9560447841444775	0.9560667758597363
642	0.13544867	0.956401817	0.9562009239187249	0.9563897614150593	0.956413156559047I	0.9563872950664092	0.9564099213234061
672	0.131767913	0.957385532	0.9571890569871824	0.9573703926193927	0.9573969413704723	0.9573685153805663	0.9573944056755324
732	0.126909613	0.959167323	0.9589799367848233	0.9591472935682428	0.9591798893261063	0.9591466357996342	0.9591764187011856
752	0.124944956	0.959713774	0.9595287417713301	0.9596917145435805	0.9597262504564333	0.9596914704517056	0.9597230638862736
762	0.123995531	0.95997913	0.9597950100777394	0.9599558386649476	0.95999133082747	0.9599558021464992	0.9599882755309913
792	0.121761979	0.960743398	0.9605633457274354	0.9607179238055088	0.9607562304415026	0.9607185116505276	0.960752972585681
812	0.120139458	0.961229519	0.9610517518866855	0.9612023074787079	0.9612424461179326	0.9612033120045522	0.9612392917537761
842	0.118042299	0.961926579	0.9617514851702419	0.9618962104905434	0.9619390325819777	0.9618978391205292	0.9619357736480628
912	0.113139882	0.963415706	0.9632478217949435	0.9633798100493296	0.9634286061512165	0.9633828822825516	0.9634255858148685
932	0.112090932	0.963809966	0.963644080386371	0.9637726333965143	0.9638230660450318	0.9637761131897101	0.9638198461347699
942	0.111552054	0.964002253	0.9638374624340769	0.9639643294243279	0.9640155691198593	0.9639680120046147	0.9640122817322361
972	0.110319452	0.964561383	0.9643996015382518	0.9645215316950216	0.964575148783067	0.9645258183415738	0.9645713271413074
1152	0.100924821	0.967444905	0.9672975609710157	0.9673931817355069	0.967459810464484	0.9674009344555994	0.9674563981093801
1382	0.091842819	0.97027538	0.9701413760167691	0.9702097394836015	0.9702904078390541	0.9702214882762551	0.9702872638676995
1500	0.088510326	0.971467435	0.9713393959507919	0.9713958362329984	0.9714828095008867	0.9714094520282464	0.9714793692244232
1632	0.086084285	0.972644414	0.9725224597112828	0.9725668633710967	0.9726602959638073	0.9725824328726311	0.972655904724115
1902	0.079766338	0.974659171	0.9745466856872055	0.9745698781908521	0.974674911826722	0.9745890504103528	0.974670716556055
2000	0.076664943	0.975287819	0.9751779198420939	0.9751943359820057	0.9753031317501037	0.9752147002336796	0.9752998221319861
2192	0.072940512	0.976394878	0.9762895869680002	0.9762938790075385	0.9764094703416387	0.9763164234684998	0.9764064214392618
2500	0.068475173	0.977895748	0.9777977144702729	0.9777851650287557	0.9779103277662224	0.9778108352322674	0.9779072342998565
2502	0.068265972	0.977904659	0.9778065875917212	0.9777939377067558	0.9779191579733574	0.9778196268812956	0.9779161832966433
2832	0.064316986	0.979231026	0.9791393278365985	0.9791114090114895	0.9792454337998905	0.9791400259590192	0.9792424413285431
3182	0.060565561	0.980405934	0.9803197283516336	0.9802779800337199	0.9804200777420188	0.9803093218388931	0.9804172369723245
3552	0.057472491	0.981453993	0.9813726880044842	0.9813183570543405	0.9814678776367772	0.9813522359916628	0.9814650352529444
3942	0.054246302	0.982394779	0.98231792549536	0.9822520986943531	0.9824084633639443	0.982288342176562	0.9824058682066104
4352	0.051648432	0.983244295	0.9831712673088749	0.9830948973819746	0.983257589478656	0.9831333472809355	0.9832550522189838
**MSE**			2.2138×10^{-6}	6.1080×10^{-8}	8.4161×10^{-8}	5.5549×10^{-8}	7.9963×10^{-8}

Approximation of Energy of Thomson Problem by the models

We present the approximation of the energy, $E(n)$, of the Thomson problems by our model in Table S2 for the convenience of the reader.

Table S2. The approximations to the Energy ($E(n)$) in Thomson problem presented by the models in this contribution.

n	E(n)	eqs. (6),(7)	eqs. (12),(13)	eqs. (14),(15)	eqs. (12),(17)	eq. (18)
2	0.5	0.5571221340532733	0.5002868352949871	0.502905838106147	0.5009296587662314	0.5102242213641713
3	1.73205081	1.7652835573318357	1.7209978671714934	1.7132867323396155	1.7206319092697941	1.727817445132325
4	3.674234616	3.728692696884749	3.6958578693141675	3.6807839235780557	3.695088496513312	3.689343614698188
5	6.4746915	6.481419346738253	6.458428912376974	6.43850347370115	6.457684602648832	6.456287799792572
6	9.985281372	10.046271760117655	10.031924961526979	10.009098322417719	10.03154493569298	10.00963780808544
7	14.4529774	14.439927430095471	14.433402685099116	14.409195984212865	14.433672279608711	14.439729125091706
8	19.67528787	19.675275079111767	19.67604273195626	19.651655827841676	19.67720998156016	19.668062278290755
9	25.75998653	25.762665173766553	25.770415758760716	25.746804469087216	25.772700656218873	25.75538914520698
10	32.71694945	32.710648372231375	32.72523972554715	32.70317099986537	32.72884005495901	32.714096099541834
11	40.59645052	40.526443921952136	40.547862606301095	40.52795464280118	40.55295793198636	40.561453795171815
12	49.16525306	49.21625320585908	49.24458607852336	49.22733810109368	49.25134063408444	49.20376650254069
13	58.85323059	58.78547867401622	58.82089160541611	58.80670640313219	58.82945647577247	58.857881597967584
14	69.30636332	69.23888199909624	69.28160368986491	69.270804982408	69.29211851236877	69.29924691818695
15	80.67024416	80.58070157548778	80.63101106721234	80.62385709597388	80.64360539112356	80.67476746281993
16	92.9116553	92.81474189149985	92.87295879796571	92.86965311247218	92.88775319277339	92.90645443791792
17	106.0504048	105.94444288354656	106.01091965760737	106.01161978263875	106.02802663154873	106.01155586533365
18	120.0844673	119.97293469404269	120.04805044007698	120.05287491707871	120.06757521191895	120.0699448315844
19	135.0894676	134.90308156082213	134.98723703647894	134.99627120089164	135.00927819167418	135.03687445359444
20	150.8815684	150.73751746415113	150.83113100839725	150.84443177199958	150.85578106264012	150.84203090765246
21	167.6416225	167.47867542104746	167.58217961167807	167.59977945304314	167.60952549925554	167.60975618641166
22	185.2875362	185.12881181254318	185.24265070392659	185.26456102227328	185.2727742042408	185.2738418993525
23	203.9301906	203.69002677707314	203.81465360365306	203.84086755622042	203.84763171636345	203.87042814489365
24	223.3470741	223.1642814520218	223.30015670880618	223.3306516256913	223.3360619858616	223.32293464754167
25	243.8127603	243.55341266342978	243.70100249393982	243.73574194459104	243.73990333512938	243.7709729144333
26	265.1333263	264.85914552988737	265.01892036661314	265.05785593710937	265.0608812840048	265.0936059941963
27	287.302615	287.0831043466518	287.2555377602096	287.29861058885155	287.3006196158686	287.27768394824244
28	310.4915423	310.2268220404427	310.4123897622447	310.45953187195266	310.4606499828571	310.4832858268685
29	334.6344399	334.2917484275701	334.49092751753057	334.54206297645686	334.5424202889467	334.59317800634386
30	359.603946	359.27925746337115	359.49252559945205	359.5475715356049	359.54730204367814	359.5563427395181
31	385.530838	385.1906536360506	385.41848850663274	385.4773559978317	385.4765968434029	385.48441936347893
32	412.2612746	412.0271776305447	412.27005641394516	412.33265127083587	412.33154210868423	412.27351664137353
33	440.2040573	439.7900113661916	440.04841028433174	440.11463374127544	440.1133161840569	440.1881954797972
34	468.904853	468.4802824945095	468.7546764299041	468.82442575619297	468.8230428883913	468.8961262244335
35	498.5698728	498.0990684292742	498.38993059627666	498.4630996381847	498.4617955896396	498.5216311936836
36	529.1224087	528.6473999696307	528.9552016323105	529.0316812948967	529.0306008659845	529.0615014706867
37	560.6188875	560.1262645676004	560.4514747978171	560.5311534740704	560.5304418058136	560.584428284981
38	593.0385036	592.5366092836406	592.8796947538569	592.9624587076803	592.9622609910448	592.9434235114056
39	626.3890092	625.8793434675346	626.2407682737243	626.3265019823308	626.3269632018038	626.3492630919928

Continued on next page

Table S2 – *Continued from previous page*

n	E(n)	eqs. (6),(7)	eqs. (12),(13)	eqs. (14),(15)	eqs. (12),(17)	eq. (18)
40	660.6752792	660.1553411965843	660.5355667072745	660.6241531677961	660.6254178750256	660.6308632239572
41	695.9167445	695.3654434986474	695.7649282266947	695.85624923116	695.8584613450216	695.8621504146369
42	732.0781075	731.5104603838289	731.9296598780154	732.0235962602945	732.0268988902418	732.0227672958559
43	769.1908468	768.5911727054979	769.0305394594315	769.1269713172827	769.1315066072578	769.1261709882535
44	807.1742633	806.608333868634	807.0683172447784	807.1671241397308	807.1730331302645	807.1138362123158
45	846.1884014	845.5626714012369	846.043717568192	846.1447787056562	846.1522012120903	846.1398980961928
46	886.1671137	885.4548884025984	885.9574402839942	886.0606346756996	886.069709180727	886.0613442773564
47	927.0592707	926.285664880572	926.8101621141527	926.9153687247597	926.9262322836979	926.9683247175055
48	968.7134557	968.0556589885443	968.6025378942035	968.7096357737157	968.7224239311216	968.6667413792748
49	1011.557183	1010.7655081715795	1011.3352017272579	1011.4440701306761	1011.45891684708	1011.4819694513552
50	1055.182315	1054.4158302301332	1055.008768054631	1055.1192865501214	1055.1363241377994	1055.105188586133
51	1099.81929	1099.0072243088086	1099.623832650677	1099.7358812173861	1099.7552402842132	1099.7439048617232
52	1145.418964	1144.5402718168082	1145.1809735485883	1145.2944326651098	1145.3162420656527	1145.3221732361358
53	1191.92229	1191.0155372860356	1191.6807519031977	1191.7955026275881	1191.8198894206826	1191.8484780602057
54	1239.361475	1238.4335691721706	1239.1237127961851	1239.239636838331	1239.2667262504785	1239.2789478526731
55	1287.772721	1286.7949006035012	1287.5103859885417	1287.62736577559	1287.6572811695803	1287.6834296989996
56	1337.094945	1336.1000500818147	1336.841286624643	1336.9592053601425	1336.99206820837	1336.9842863496306
57	1387.383228	1386.349522139222	1387.1169158918688	1387.23565760919	1387.2715874711935	1387.2337053621766
58	1438.618252	1437.5438079544117	1438.337761639299	1438.457211249861	1438.4963257536604	1438.4886818811801
59	1490.773335	1489.6833859315068	1490.5042989587068	1490.624342295467	1490.666757122319	1490.6292686155855
60	1543.830401	1542.768722244387	1543.6169907307406	1543.7375145873723	1543.7833434596077	1543.741081893557
61	1597.941831	1596.8002713490816	1597.6762881389332	1597.7971803050716	1597.8465349767027	1597.8414393585522
62	1652.90941	1651.7784764666055	1652.682631153938	1652.8037804468336	1652.8567706966633	1652.7646024163569
63	1708.879681	1707.7037700383887	1708.6364489901655	1708.757745283059	1708.8144789100395	1708.768604072116
64	1765.802578	1764.5765741562745	1765.5381605368157	1765.6594947843128	1765.720077604935	1765.6873642653363
65	1823.66796	1822.3973009688775	1823.3881747651174	1823.5094390258223	1823.573974873332	1823.563106609358
66	1882.441525	1881.1663530659507	1882.186891113441	1882.3079785700772	1882.3765692953377	1882.3054298678426
67	1942.122699	1940.8841238422663	1941.934699851804	1942.0555048290348	1942.1282503028717	1942.0320707089063
68	2002.874701	2001.5509978423954	2002.6319824271623	2002.7524004073048	2002.829398524186	2002.74345247191
69	2064.533483	2063.167351087653	2064.279111790777	2064.3990394275816	2064.4803861104956	2064.406161780367
70	2127.100903	2125.7335513863814	2126.8764527088274	2126.995787839485	2127.081577045898	2126.954583304636
71	2190.649905	2189.249958628645	2190.424362057362	2190.5430037128835	2190.6333274416647	2190.547647488806
72	2255.00119	2253.716925066331	2254.923189102585	2255.0410375166903	2255.135985815901	2254.914688737217
73	2320.633885	2319.1347955795704	2320.373275767405	2320.4902323840333	2320.5898933594995	2320.59714172051
74	2387.072982	2385.5039079303306	2386.7749568850936	2386.890924364662	2386.9953841892325	2386.94921928472
75	2454.369688	2452.8245930039566	2454.1285604408536	2454.2434426653535	2454.352785588785	2454.314238571701
76	2522.674873	2521.0971750393924	2522.434407802019	2522.5481098790506	2522.6624182384376	2522.5695058155698
77	2591.850151	2590.3219718487544	2591.6928139375705	2591.8052422033975	2591.9245964340953	2591.733049618307
78	2662.046476	2660.499295026883	2661.90408762759	2662.015149649302	2662.139628296278	2661.891063643551

Continued on next page

Table S2 – *Continued from previous page*

n	E(n)	eqs. (6),(7)	eqs. (12),(13)	eqs. (14),(15)	eqs. (12),(17)	eq. (18)
79	2733.248356	2731.6294501514467	2733.068531663249	2733.1781362400952	2733.307815969661	2733.1649064244107
80	2805.355875	2803.712736974149	2805.186443037863	2805.294500201825	2805.4294558137085	2805.229178599137
81	2878.52283	2876.7494496035333	2878.2581131295233	2878.364534145197	2878.504838584897	2878.434534498359
82	2952.569676	2950.739876679858	2952.2838278757804	2952.3885252396153	2952.534249611014	2952.3748606196723
83	3027.528488	3025.684301542472	3027.263867940812	3027.366755379765	3027.517968957953	3027.433428377099
84	3103.465123	3101.5830023901167	3103.1985088754914	3103.2995013451364	3103.4562715894303	3103.3671612334515
85	3180.361443	3178.4362524345083	3180.0880212707343	3180.187034952886	3180.349427519991	3180.2327111249897
86	3258.211608	3256.244320047579	3257.9326709044917	3258.029623204366	3258.197701961674	3258.0450248494926
87	3337.000749	3335.007468902705	3336.732718882706	3336.8275284256747	3337.001355464662	3336.8302894645517
88	3416.720196	3414.7259581102276	3416.48842177457	3416.581008402526	3416.7606440522363	3416.5513944403015
89	3497.439017	3495.4000423475613	3497.200031742354	3497.2903165097355	3497.475819350323	3497.298469124311
90	3579.091223	3577.029971984177	3578.867796666105	3578.9557018355963	3579.1471287119093	3578.9272255565193
91	3661.713701	3659.615993201695	3661.4919602634495	3661.577409301397	3661.774815336591	3661.5497894561368
92	3745.291637	3743.1583481093494	3745.072762204769	3745.1556797763255	3745.359118385487	3745.118898838324
93	3829.844338	3827.657274855044	3829.610438223953	3829.6907501879878	3829.900273091752	3829.6897446386147
94	3915.309268	3913.11300773221	3915.105220224967	3915.182853628752	3915.398510866907	3915.1087402048847
95	4001.771676	3999.525777282676	4001.5573363844137	4001.632219458114	4001.854059403181	4001.587247970157
96	4089.15401	4086.89581039573	4088.9670112503	4089.0390734012817	4089.267142772053	4088.972833854834
97	4177.5336	4175.2233304035635	4177.334465837177	4177.403637644157	4177.637981519187	4177.367649894971
98	4266.822464	4264.508557173253	4266.659917717821	4266.726130924866	4266.96679275591	4266.670045040557
99	4357.139161	4354.751707195453	4356.943581111625	4357.00676862202	4357.253790247415	4356.957851688035
100	4448.350635	4445.952993669935	4448.185666969839	4448.245762839844	4448.499184497805	4448.166433662738
101	4540.590052	4538.1126265881285	4540.386383057814	4540.44332249031	4540.703182832161	4540.4104608245025
102	4633.736568	4631.230812812796	4633.545934034372	4633.599653372415	4633.865989475743	4633.532170692923
103	4727.836618	4725.3077561549535	4727.664521528443	4727.714958248742	4727.98780563045	4727.655530336951
104	4822.876524	4820.343657448176	4822.742344213073	4822.78943691939	4823.068829548668	4822.689080798669
105	4919.00064	4916.3387146204	4918.779597876931	4918.823286293428	4919.10925660463	4918.7265574984285
106	5015.984598	5013.293122763314	5015.776475493421	5015.816700457945	5016.109279363364	5015.743410249235
107	5113.95355	5111.207074199459	5113.733167287488	5113.769870744822	5114.069087647361	5113.763184228818
108	5212.81351	5210.080758547123	5212.6498608002385	5212.682985795302	5212.988868601048	5212.649696870509
109	5312.735078	5309.914362783125	5312.526740951445	5312.556231622478	5312.868806753161	5312.561471593993
110	5413.549293	5410.708071303588	5413.363990100035	5413.389791671754	5413.709084077103	5413.257700500776
111	5515.293216	5512.462065982754	5515.161788102643	5515.183846879378	5515.509880049362	5515.089643210843
112	5618.044884	5615.176526229958	5617.920312370309	5617.9385757291275	5618.27137170609	5617.786993250901
113	5721.824979	5718.851629044799	5721.639737923389	5721.654154307222	5721.993733697891	5721.589644314963
114	5826.521574	5823.487549070612	5826.320237444757	5826.330756355516	5826.677138342917	5826.283227561284
115	5932.181289	5929.084458646287	5931.961981331376	5931.968553323071	5932.321755678304	5932.013885216081
116	6038.815591	6035.642527856505	6038.565137744264	6038.567714416139	6038.927753510057	6038.610328220585
117	6146.342445	6143.161924580455	6146.129872656975	6146.128406646641	6146.495297461407	6146.085995665083

Continued on next page

Table S2 – *Continued from previous page*

n	E(n)	eqs. (6),(7)	eqs. (12),(13)	eqs. (14),(15)	eqs. (12),(17)	eq. (18)
118	6254.877026	6251.642814539093	6254.656349902598	6254.650794879193	6255.02455101971	6254.618067549565
119	6364.347317	6361.085361340992	6364.144731219368	6364.135041876723	6364.5156755819635	6364.1148931826865
120	6474.756326	6471.489726526838	6474.595176294918	6474.581308344756	6474.968830498963	6474.506509776398
121	6586.121949	6582.856069612635	6586.007842809247	6585.9897529743885	6586.384173118168	6585.962787168869
122	6698.3745	6695.184548131637	6698.382886476422	6698.360532484036	6698.761858825324	6698.162261017797
123	6811.827227	6808.475317675097	6811.7204610850895	6811.69380165996	6812.102041084878	6811.7391887605345
124	6926.169972	6922.728531931838	6926.020718537824	6925.989713395645	6926.404871479244	6925.911829541201
125	7041.473266	7037.944342726709	7041.283808889358	7041.2484187300715	7041.670499746954	7041.2313472360565
126	7157.669222	7154.122900057959	7157.509880383742	7157.4700668849	7157.899073819731	7157.406273275373
127	7274.819507	7271.264352133576	7274.699079490458	7274.654805300626	7275.090739858524	7274.57947212104
128	7393.007444	7389.368845406616	7392.851550939539	7392.802779671737	7393.245642288563	7392.72340169273
129	7512.10732	7508.436524609568	7511.967437755728	7511.914133980899	7512.363923833424	7511.834299969889
130	7632.167375	7628.467532787774	7632.046881291686	7631.9890105322265	7632.445725548188	7631.904257976484
131	7753.205163	7749.462011331969	7753.090021260328	7753.027549983636	7753.491186851685	7752.982101305897
132	7875.045339	7871.42010000992	7875.096995766273	7875.02989137835	7875.50044555789	7874.780642662572
133	7998.179214	7994.341936997252	7998.067941336456	7997.996172175552	7998.473637906464	7998.046848351459
134	8122.089723	8118.227658907439	8122.002992949939	8121.926528280237	8122.410898592505	8121.918165351611
135	8246.909484	8243.077400821026	8246.902284066937	8246.821094072286	8247.312360795502	8246.696872991422
136	8372.743302	8368.891296314081	8372.765946657084	8372.680002434767	8373.178156207547	8372.505157678177
137	8499.534499	8495.669477485913	8499.594111226967	8499.503384781534	8500.008415060807	8499.268354762944
138	8627.406386	8623.412074986094	8627.386906846961	8627.291371084091	8627.803266154304	8627.29593932724
139	8756.227052	8752.119218040772	8756.144461177375	8756.044089897785	8756.56283688	8756.129057921911
140	8885.980607	8881.791034478341	8885.866900493937	8885.76166838735	8886.287253248229	8885.777730337499
141	9016.615347	9012.427650754455	9016.554349712635	9016.444232351781	9016.976639912482	9016.350815701062
142	9148.27158	9144.029191976419	9148.206932413947	9148.091906248626	9148.631120193595	9148.024536427305
143	9280.839854	9276.59578192699	9280.824770866464	9280.704813217644	9281.250816103306	9280.635473809307
144	9414.371795	9410.127543087578	9414.407986049939	9414.283075103902	9414.835848367258	9414.108716024613
145	9548.928833	9544.624596660897	9548.956697677759	9548.826812480307	9549.386336447438	9548.675083763022
146	9684.381831	9680.08706259305	9684.47102421889	9684.336144669589	9684.90239856405	9684.126334425542
147	9820.932374	9816.515059595105	9820.95108291929	9820.81118976576	9821.384151716898	9820.780724142647
148	9958.406008	9953.90870516414	9958.396989822784	9958.252064655047	9958.831711706218	9958.114378087119
149	10096.85991	10092.268115603794	10096.80885979149	10096.658885036359	10097.24519315305	10096.627762026043
150	10236.19644	10231.593406044336	10236.186806525726	10236.031765441236	10236.624709519101	10235.944289526633
151	10376.57147	10371.88469046226	10376.530942583453	10376.370819253354	10376.97037312616	10376.372971519757
152	10517.86759	10513.142081699434	10517.841379399291	10517.67615872758	10518.282295175059	10517.674127343667
153	10660.08275	10655.365691481798	10660.118227303066	10659.947895008565	10660.560585764188	10659.783178323134
154	10803.37243	10798.555630437631	10803.361595537946	10803.186138148933	10803.805353907592	10803.149316727822
155	10947.57469	10942.712008115419	10947.571592278171	10947.390997127048	10948.01670755265	10947.371435486597
156	11092.79831	11087.8349330013	11092.748324646345	11092.562579864372	11093.194753597354	11092.601924875955

Continued on next page

Table S2 – *Continued from previous page*

n	E(n)	eqs. (6),(7)	eqs. (12),(13)	eqs. (14),(15)	eqs. (12),(17)	eq. (18)
157	11238.90304	11233.924512536123	11238.891898730395	11238.700993242446	11239.339597907207	11238.648205099524
158	11385.99019	11380.98085313213	11386.00241960009	11385.80634311948	11386.451345331725	11385.705780280367
159	11534.02396	11529.004060189267	11534.079991323246	11533.87873434658	11534.530099720589	11533.759487319683
160	11683.05481	11677.994238111125	11683.124716981525	11682.918270783617	11683.575963939433	11682.775064468127
161	11833.08474	11827.951490320549	11833.136698685928	11832.925055314747	11833.589039885283	11832.83483504104
162	11984.05034	11978.875919274888	11984.116037591926	11983.899189863594	11984.569428501662	11983.779361123605
163	12136.01305	12130.76762648093	12136.062833914266	12135.840775408104	12136.517229793375	12135.768092342505
164	12288.93011	12283.626712509513	12288.977186941464	12288.749911995077	12289.432542840947	12288.660640317967
165	12442.80445	12437.453277009812	12442.859195049978	12442.62669875439	12443.315465814798	12442.511576861856
166	12597.64907	12592.247418723337	12597.708955718093	12597.471233912926	12598.166095989067	12597.352942358817
167	12753.46942	12748.00923549763	12753.526565539501	12753.283614808188	12753.984529755178	12753.141365210782
168	12910.21267	12904.738824299666	12910.312120236595	12910.063937901647	12910.770862635109	12909.861649897055
169	13068.00645	13062.436281228991	13068.065714673487	13067.812298791803	13068.525189294374	13067.648925864942
170	13226.68108	13221.101701530575	13226.787442868768	13226.528792226982	13227.247603554759	13226.318440424979
171	13386.35593	13380.7351796074	13386.477398007979	13386.213512117847	13386.938198406762	13386.016518079445
172	13547.01811	13541.336809032795	13547.135672455846	13546.866551549687	13547.597066021812	13546.762519948337
173	13708.63524	13702.906682562523	13708.76235776826	13708.48800279443	13709.22429776421	13708.396667008146
174	13871.1871	13865.444892146603	13871.357544704006	13871.077957322408	13871.819984202846	13870.872843944895
175	14034.78131	14028.951528940928	14034.921323236267	14034.63650581392	14035.384215122669	14034.472548750658
176	14199.35479	14193.426683318614	14199.453782563893	14199.163738170517	14199.91707953593	14199.063804792544
177	14364.83755	14358.870444881148	14364.955011122433	14364.659743526096	14365.418665693207	14364.42735709529
178	14531.30955	14525.282902469302	14531.42509659497	14531.124610257766	14531.889061094194	14530.941199378502
179	14698.75458	14692.664144173841	14698.864125922726	14698.558425996487	14699.328352498298	14698.458169808144
180	14867.09993	14861.014257346014	14867.272185315462	14866.96127763752	14867.736625935016	14866.769633188784
181	15036.46725	15030.333328607841	15036.649360261677	15036.333251350656	15037.113966714109	15036.15497189796
182	15206.73061	15200.621443862212	15206.995735538612	15206.67443259026	15207.460459435577	15206.367706341825
183	15378.16657	15371.87868830277	15378.311395222054	15377.984906105115	15378.776187999461	15377.916275865568
184	15550.42145	15544.10514642363	15550.596422695953	15550.264755948081	15551.06123561542	15550.045802288358
185	15723.72007	15717.300902028888	15723.850900661866	15723.514065485562	15724.315684812165	15723.370577909393
186	15897.89744	15891.466038241973	15898.074911148198	15897.732917406804	15898.53961744669	15897.585329797474
187	16072.9752	16066.600637514806	16073.268535519306	16072.92139373301	16073.733114713319	16072.67143216839
188	16249.22269	16242.704781636789	16249.431854484386	16249.07957582629	16249.89625715263	16248.87443268768
189	16426.37193	16419.77855174364	16426.56494810624	16426.20754439844	16427.02912466015	16426.021053506942
190	16604.42833	16597.822028326038	16604.66789580983	16604.30537951956	16605.131796494927	16604.208017518904
191	16783.45222	16776.835291238152	16783.74077639074	16783.373160626503	16784.20435128795	16783.462487350338
192	16963.33839	16956.818419705956	16963.783668023396	16963.410966531195	16964.246867050384	16963.03280744098
193	17144.56475	17137.771492335447	17144.796648269235	17144.418875428775	17145.259421181672	17144.490723977167
194	17326.61614	17319.694587120666	17326.77979408463	17326.3969649056	17327.242090477488	17326.29416694953
195	17509.4893	17502.58778145162	17509.733181828742	17509.3453119471	17510.19495113755	17509.19662682775

Continued on next page

Table S2 – *Continued from previous page*

n	E(n)	eqs. (6),(7)	eqs. (12),(13)	eqs. (14),(15)	eqs. (12),(17)	eq. (18)
196	17693.46055	17686.451152122027	17693.656887271194	17693.263992945507	17694.11807877329	17693.17735021528
197	17878.34017	17871.284775336928	17878.550985599617	17878.153083707424	17879.011548415365	17878.115164805196
198	18064.26219	18057.088726720183	18064.415551427075	18064.012659461263	18064.875434521084	18063.879915233025
199	18251.0825	18243.86308132181	18251.250658799316	18250.842794864584	18251.70981098164	18250.74474117257
200	18438.84272	18431.607913625227	18439.05638120196	18438.643564011247	18439.514751129278	18438.465732328998
201	18627.59124	18620.32329755431	18627.8327915675	18627.415040438496	18628.290327744282	18627.50557008798
202	18817.20471	18810.009306480395	18817.579962282212	18817.157297133865	18818.036613061868	18816.946837527918
203	19007.98119	19000.66601322911	19008.29796519294	19007.87040654203	19008.753678778972	19007.794497467337
204	19199.54077	19192.293490087115	19199.98687161377	19199.55444057147	19200.44159606088	19199.20705633186
205	19392.36916	19384.891808808716	19392.646752332566	19392.209470601076	19393.100435547767	19392.12979330625
206	19585.95585	19578.46104062236	19586.27767761742	19585.83556748659	19586.730267361145	19585.706244001478
207	19780.6569	19773.001256237036	19780.87971722298	19780.432801566996	19781.331161110156	19780.259428245892
208	19976.20326	19968.51252584856	19976.452940396666	19976.001242670754	19976.903185897776	19975.884357207495
209	20172.75468	20164.994919145738	20172.99741588479	20172.54096012195	20173.446410326953	20172.399691278326
210	20370.25162	20362.448505316457	20370.513211938578	20370.052022746335	20370.96090250657	20369.91572157781
211	20568.7406	20560.87335305364	20569.000396320083	20568.53449887727	20569.446730057367	20568.604133949222
212	20768.05309	20760.26953056114	20768.459036307977	20767.988456361563	20768.90396011774	20767.537865652415
213	20968.61203	20960.637105559505	20968.889198703302	20968.413962565242	20969.33265934945	20968.336041225866
214	21169.9104	21161.976145291646	21170.29094983508	21169.81108437916	21170.732893943226	21169.588896199166
215	21372.34878	21364.286716528455	21372.664355565834	21372.179888224593	21373.1047296243	21372.1034728797
216	21575.59637	21567.568885574277	21576.009481297053	21575.520440058703	21576.448231657825	21575.27005054977
217	21779.85609	21771.822718272324	21780.326391974533	21779.832805379905	21780.763464854226	21779.411271743902
218	21985.26396	21977.048280010007	21985.615152093644	21985.117049233162	21986.050493574447	21984.95879753835
219	22191.48547	22183.24563572416	22191.875825704512	22191.373236215208	22192.30938173513	22191.2556077951
220	22398.6556	22390.41484990618	22399.10847641712	22398.60143047966	22399.540192813707	22398.349280702656
221	22606.88155	22598.555986607123	22607.31316740633	22606.801695742055	22607.742989853385	22606.641681445282
222	22816.02558	22807.669109442675	22816.489961416806	22815.974095284833	22816.9178354681	22815.751991666955
223	23026.16587	23017.754281598063	23026.6389207679	23026.118691962205	23027.064791847355	23025.879566398682
224	23237.24487	23228.811565832897	23237.7601073584	23237.23554820497	23238.18392076099	23236.86542093705
225	23449.43647	23440.841024485904	23449.853582671272	23449.32472602524	23450.275283563882	23449.275637688243
226	23662.51111	23653.842719479642	23662.919407778278	23662.386287021094	23663.338941200578	23662.160252757254
227	23876.57689	23867.816712325093	23876.957643344536	23876.420292381197	23877.37495420984	23876.185282751605
228	24091.57899	24082.7630641262	24091.968349633025	24091.42680288927	24092.38338272913	24091.236162820172
229	24307.59931	24298.68183558435	24307.951586508996	24307.40587892857	24308.36428649902	24307.30609780221
230	24524.48536	24515.57308700275	24524.907413444336	24524.357580486252	24525.317724867546	24524.115582890507
231	24742.38248	24733.436878290795	24742.835889521855	24742.28196715769	24743.243756794483	24742.109348866263
232	24961.25233	24952.27326896831	24961.737073439497	24961.17909815072	24962.142440855554	24960.88744775247
233	25181.0574	25172.082318169756	25181.611023514524	25181.04903228981	25182.013835246602	25180.663123459344
234	25401.9318	25392.864084648387	25402.4577976876	25401.891828020205	25402.85799778765	25401.661514349467

Continued on next page

Table S2 – *Continued from previous page*

n	E(n)	eqs. (6),(7)	eqs. (12),(13)	eqs. (14),(15)	eqs. (12),(17)	eq. (18)
235	25623.76315	25614.61862678031	25624.27745352681	25623.70754341196	25624.67498592695	25623.509235696012
236	25846.50055	25837.346002568513	25847.070048231675	25846.496236163952	25847.464856744937	25846.17931231085
237	26070.367	26061.04626964682	26070.835638637032	26070.257963607804	26071.227666958144	26070.045310114707
238	26295.12604	26285.71948528379	26295.574281216905	26294.992782711783	26295.96347292305	26294.79579146877
239	26520.87411	26511.365706386565	26521.286032088305	26520.700750084605	26521.672330639867	26520.59510711651
240	26747.50821	26737.984989504635	26747.970947014994	26747.381921979202	26748.354295756297	26747.138556820228
241	26975.19029	26965.577390833605	26975.629081411134	26975.036354296455	26976.00942357119	26974.877697097436
242	27203.79927	27194.142966218835	27204.260490344976	27203.66410258882	27204.637769038192	27203.4429892397
243	27433.36735	27423.68177115908	27433.86522854239	27433.265222063965	27434.239386769306	27433.066975679212
244	27663.90512	27654.19386081006	27664.443350390447	27663.839767588277	27664.814331038437	27663.515352314593
245	27895.54073	27885.679289987984	27895.99490994087	27895.38779369042	27896.362655784847	27895.23111514991
246	28128.05145	28118.138113172994	28128.519960913472	28127.90935456474	28128.884414616587	28127.6416271352
247	28361.53276	28351.570384512623	28362.018556699546	28361.40450407469	28362.379660813876	28361.23992286254
248	28596.0521	28585.976157825135	28596.490750365185	28595.873295756173	28596.84844733242	28595.648700892136
249	28831.47391	28821.355486602868	28831.93659465459	28831.315782820853	28832.29082680671	28831.079960135576
250	29067.87769	29057.70842401549	29068.3561419933	29067.732018159426	29068.70685155323	29067.602436917634
251	29305.23387	29295.035022913256	29305.74944449138	29305.122054344807	29306.09657357367	29305.058831542243
252	29543.52285	29533.33533583017	29544.116553946584	29543.485943635325	29544.460044558058	29543.21910630169
253	29782.91736	29772.60941498714	29783.457521847464	29782.823737977837	29783.797315887863	29782.59920624532
254	30023.22286	30012.85731229507	30023.77239937642	30023.13548901081	30024.10843863905	30023.15832263954
255	30264.42426	30254.079079357918	30265.061237412727	30264.42124806735	30265.3934635851	30264.167491727552
256	30506.68753	30496.27476747571	30507.324086535536	30506.681066178233	30507.65244119999	30506.39052158196
257	30749.94143	30739.4444276475	30750.560997026776	30749.914994074825	30750.8854216611	30749.48454815743
258	30994.21359	30983.588110574325	30994.77201887408	30994.123082192007	30995.092454852136	30993.8609826724
259	31239.44229	31228.705866662076	31239.957201773632	31239.305380671074	31240.27359036597	31239.108133461188
260	31485.57819	31474.797746024353	31486.116595133008	31485.461939362543	31486.42887750746	31485.089544051298
261	31732.74279	31721.86379848529	31733.250248073924	31732.59280782897	31733.558365296212	31732.350039846006
262	31980.85183	31969.904073582322	31981.358209435013	31980.6980353477	31981.662102469338	31980.37618772178
263	32229.91489	32218.91862056892	32230.44052777452	32229.77767091361	32230.740137484157	32229.47727246109
264	32479.90813	32468.907488417306	32480.497251372974	32479.83176324177	32480.792518520844	32479.422665118425
265	32731.01238	32719.87072582111	32731.528428235826	32730.860360770137	32731.819293485085	32730.57245737543
266	32982.98362	32971.80838119801	32983.534106096056	32982.86351166212	32983.82051001066	32982.56740822499
267	33235.96207	33224.72050269231	33236.514332416744	33235.84126380921	33236.796215461996	33235.51907634941
268	33489.87447	33478.60713817754	33490.46915439357	33489.79366483351	33490.74645693672	33489.50654151146
269	33744.80074	33733.468335258935	33745.39861895738	33744.72076209023	33745.67128126816	33744.38239419832
270	34000.6485	33989.30414127596	34001.30277277658	34000.622602670235	34001.57073502774	34000.59793174433
271	34257.44173	34246.1146033048	34258.18166225964	34257.49923340241	34258.444864527504	34257.570842802066
272	34515.19328	34503.89976816072	34516.03533355743	34515.35070085612	34516.29371582247	34514.85841125792
273	34774.25786	34762.65968240055	34774.86383256567	34774.17705134366	34775.11733471296	34773.778748912126

Continued on next page

Table S2 – *Continued from previous page*

n	E(n)	eqs. (6),(7)	eqs. (12),(13)	eqs. (14),(15)	eqs. (12),(17)	eq. (18)
274	35034.03345	35022.394392324975	35034.66720492722	35033.97833092248	35034.915766747035	35033.59399269991
275	35294.89406	35283.10394398095	35295.44549603437	35294.75458539763	35295.68905722272	35294.45864689728
276	35556.63332	35544.788383163934	35557.19875103122	35556.505860324	35557.43725119029	35556.281049185265
277	35819.34079	35807.447755420246	35819.927014815825	35819.232201008606	35820.16039345458	35818.93232827709
278	36083.03688	36071.08210604925	36083.63033204251	36082.933652512824	36083.85852857713	36082.67155281017
279	36347.65844	36335.69148010563	36348.308747123985	36347.61025965459	36348.531700878455	36347.27420080309
280	36613.26958	36601.27592240154	36613.96230423359	36613.26206701062	36614.179954440115	36613.23193333907
281	36879.8056	36867.83547750881	36880.591047307404	36879.88911891852	36880.80333310695	36879.93687526445
282	37147.29442	37135.370189761066	37148.19502004634	37147.49145947897	37148.40188048913	37146.90317880156
283	37416.07573	37403.880103255855	37416.774265918284	37416.069132557765	37416.97563996428	37415.82418056776
284	37685.6898	37673.365261856714	37686.32882816013	37685.622181787934	37686.524654679495	37685.32654981113
285	37956.23944	37943.82570919526	37956.8587497798	37956.15065057179	37957.04896755342	37956.10948729094
286	38227.67331	38215.261488673204	38228.364073558296	38227.654582082934	38228.54862127822	38227.24937534468
287	38500.13432	38487.672643464364	38500.84484205167	38500.13401926828	38501.023658321596	38499.77559856509
288	38773.58127	38761.059216516674	38774.301097593	38773.589004850015	38774.474120928724	38773.24317428335
289	39048.0492	39035.42125055413	39048.73288229431	39048.01958132757	39048.900051124205	39047.694039955735
290	39323.41005	39310.75878807872	39324.14023804848	39323.4257909795	39324.301490713966	39323.324850252495
291	39599.71183	39587.071871372355	39600.523206531194	39599.80767586551	39600.67848128716	39599.40021489022
292	39877.008	39864.36054249876	39877.881829202735	39877.16527782815	39878.03106421804	39876.67837647064
293	40155.34908	40142.624843305355	40156.21614730991	40155.49863849486	40156.35928066776	40155.05295818606
294	40434.76231	40421.86481542507	40435.526201887806	40434.8077992797	40435.66317158627	40434.54041537878
295	40715.14173	40702.08050027821	40715.81203376166	40715.09280138519	40715.94277771404	40714.9253764736
296	40996.41391	40983.271939074206	40997.07368354857	40996.35368580412	40997.1981395839	40996.03423017818
297	41278.70329	41265.43917281348	41279.31119165933	41278.590493321324	41279.42929752276	41278.35964326701
298	41561.92078	41548.582242289114	41562.52459830012	41561.80326451541	41562.63629165337	41561.673595953995
299	41846.08118	41832.701188088664	41846.71394347426	41845.992039760495	41846.81916189602	41845.750770350714
300	42131.26413	42117.79605059584	42131.87926698385	42131.156859227944	42131.97794797025	42131.02919089716
301	42417.35661	42403.86686999222	42418.02060843156	42417.297762888025	42418.11268939652	42417.013945757084
302	42704.49375	42690.91368625892	42705.138007222165	42704.414790511626	42705.22342549787	42704.18134600402
303	42992.52895	42978.936539178285	42993.23150256429	42992.50798167186	42993.31019540156	42992.413776074936
304	43281.51336	43267.93546833548	43282.301133471956	43281.577375745714	43282.37303804067	43281.17690609568
305	43571.55044	43557.91051312018	43572.34693876625	43571.62301191569	43572.41199215574	43571.26749338732
306	43862.56979	43848.861712728096	43863.368957076855	43862.64492917136	43863.42709629633	43862.05326553326
307	44154.54771	44140.789106162636	44155.36722684364	44154.643166310976	44155.41838882255	44154.29318941865
308	44447.45305	44433.69273223641	44448.34178631824	44447.61776194302	44448.38590790667	44447.111659576396
309	44741.56429	44727.57262957285	44742.2926735655	44741.56875448773	44742.3296915346	44741.44541291104
310	45036.49855	45022.42883660766	45037.2199264651	45036.49618217865	45037.249777507444	45036.22704013397
311	45332.40212	45318.26139159037	45333.123582712964	45332.400083064116	45333.146203442935	45332.02607022126
312	45629.31382	45615.07033258589	45630.00367982281	45629.28049500877	45630.01900677698	45628.993630538425

Continued on next page

Table S2 – *Continued from previous page*

n	E(n)	eqs. (6),(7)	eqs. (12),(13)	eqs. (14),(15)	eqs. (12),(17)	eq. (18)
313	45927.19254	45912.85569747589	45927.86025512754	45927.137455694996	45927.86822476504	45926.819946215306
314	46225.97975	46211.61752396032	46226.69334578075	46225.97100262443	46226.69389448367	46225.59615577798
315	46525.82567	46511.35584955886	46526.502988758126	46525.78117311934	46526.49605283187	46525.34894710385
316	46826.58842	46812.070711612345	46827.289220858875	46826.568004324115	46827.274736532534	46826.31074291889
317	47128.31033	47113.76214728417	47129.05207870712	47128.331533206605	47129.02998213383	47127.707965006164
318	47431.056	47416.43019356171	47431.791598753276	47431.07179655959	47431.76182601059	47430.73191485158
319	47734.81375	47720.0748872577	47735.50781727544	47734.78883100208	47735.47030436568	47734.67096407344
320	48039.46895	48024.696265011575	48040.20077038071	48039.48267298073	48040.15545323134	48039.155504101356
321	48345.16093	48330.29436329088	48345.870494006565	48345.1533587712	48345.81730847053	48345.06989060983
322	48651.76219	48636.86921839256	48652.517023922155	48651.80092447942	48652.45590577823	48651.36772146066
323	48959.40034	48944.42086644432	48960.140395729635	48959.42540604299	48960.07128068279	48959.35330729494
324	49267.92503	49252.949343405926	49268.74064486544	49268.02683923242	49268.663468547165	49267.4463494112
325	49577.57668	49562.45468507049	49578.31780660157	49577.60525965245	49578.232504570246	49577.202163915215
326	49888.08821	49872.936927065755	49888.87191604689	49888.16070274334	49888.7784237881	49888.0187842568
327	50199.5714	50184.39610485539	50200.40300814833	50199.6932037821	50200.30126107522	50199.24089046207
328	50512.07304	50496.83225374022	50512.91111769216	50512.20279788376	50512.80105114577	50511.752179089286
329	50825.60602	50810.245408859475	50826.396279305234	50825.6895200026	50826.27782855482	50825.371384769925
330	51140.05136	51124.635605192016	51140.85852745615	51140.153404933364	51140.73162769951	51139.78566952663
331	51455.47545	51440.002877557556	51456.29789645648	51455.594487312504	51456.16248282032	51455.10802518072
332	51771.91012	51756.34726061787	51772.714420462	51772.01280161933	51772.57042800222	51771.62980919133
333	52089.25512	52073.66878887797	52090.10813347379	52089.40838217722	52089.955497175804	52088.83317328753
334	52407.72814	52391.96749668729	52408.47906933945	52407.78126315481	52408.31772411854	52407.22170324021
335	52727.06633	52711.24341824087	52727.82726175423	52727.13147856709	52727.657142455835	52726.84487984667
336	53047.4514	53031.49658758046	53048.15274426217	53047.45906227663	53047.97378566223	53047.22565760028
337	53368.7548	53352.72703859572	53369.455550257255	53368.76404799468	53369.26768706248	53368.7070564941
338	53691.00797	53674.93480502532	53691.735712984475	53691.04646928226	53691.538879832704	53690.87988797513
339	54014.22998	53998.11992045804	54014.99326554099	54014.30635955133	54014.78739700147	54013.87120928991
340	54338.49447	54322.28241833392	54339.228240877186	54338.54375206586	54339.01327145089	54338.06274432715
341	54663.71287	54647.4223319453	54664.44067179777	54663.75867994293	54664.21653591768	54663.38905635186
342	54989.84685	54973.53969443799	54990.63059096281	54989.95117615378	54990.397222994274	54989.41895508123
343	55317.00707	55300.634538812235	55317.798030888865	55317.12127352493	55317.55536512982	55317.00065011698
344	55645.09054	55628.706897923854	55645.94302394996	55645.26900473919	55645.69099463127	55644.651801678876
345	55974.27822	55957.75680448527	55975.06560237864	55974.394402336715	55974.804143664434	55973.94034974235
346	56304.3666	56287.78429106652	56305.16579826706	56304.497498716046	56304.89484425493	56303.99658039637
347	56635.43654	56618.78939009634	56636.24364356789	56635.578326135146	56635.96312828925	56635.31646365675
348	56967.47244	56950.77213386315	56968.29917009544	56967.636916712414	56968.009027515785	56967.23537135156
349	57300.56755	57283.73255451604	57301.332409526534	57300.67330242762	57301.03257354578	57300.228509351036
350	57634.59637	57617.670684065844	57635.343393401636	57634.68751512301	57635.03379785432	57634.21466668716
351	57969.62522	57952.58655438604	57970.33215312571	57969.67958650422	57970.01273178132	57969.40443502473

Continued on next page

Table S2 – *Continued from previous page*

n	E(n)	eqs. (6),(7)	eqs. (12),(13)	eqs. (14),(15)	eqs. (12),(17)	eq. (18)
352	58305.58445	58288.480197213794	58306.29871996923	58305.64954814126	58305.969406532495	58305.36437912538
353	58642.52554	58625.351644150884	58643.243125069144	58642.59743146946	58642.903853180294	58642.460118470866
354	58980.412	58963.200926664715	58981.16539942981	58980.52326779049	58980.816102664874	58980.42528165562
355	59319.27499	59302.02807608919	59320.06557392397	59319.42708827324	59319.706185794996	59319.30009521311
356	59659.10575	59641.83312362575	59659.94367929359	59659.30892395476	59659.57413324898	59658.81616964278
357	59999.92297	59982.616100344196	60000.79974615087	60000.16880574125	60000.41997557564	59999.48374170528
358	60341.83094	60324.37703718371	60342.633804979145	60342.006764408885	60342.24374319514	60341.44005315135
359	60684.75912	60667.11596495369	60685.44588613372	60684.82283060478	60685.04546639995	60684.648217713846
360	61028.5522	61010.83291433473	61029.23601984282	61028.61703484792	61028.82517535569	61028.45052940803
361	61373.30606	61355.52791587943	61374.00423620849	61373.389407529954	61373.58290010207	61372.9399088636
362	61719.05188	61701.20100001335	61719.75056520741	61719.13997891617	61719.31867055368	61719.02054345085
363	62065.74003	62047.85219703588	62066.4750366918	62065.86877914633	62066.03251650094	62065.74850451451
364	62413.41193	62395.48153712107	62414.17768039029	62413.57583823551	62413.72446761092	62413.45396719692
365	62762.06129	62744.08905031852	62762.858525908734	62762.26118607503	62762.39455342817	62762.08953576794
366	63111.68591	63093.67476655426	63112.51760273108	63111.92485243323	63112.04280337559	63111.70412650472
367	63462.2809	63444.23871563153	63463.1549402202	63462.566866956324	63462.66924675526	63461.88187861898
368	63813.96527	63795.78092723165	63814.770567618725	63814.18725916928	63814.273912749246	63814.089974362134
369	64166.51679	64148.30143091489	64167.36451404981	64166.78605847659	64166.85683042045	64166.66820568825
370	64520.04716	64501.80025612122	64520.936808518054	64520.363294163115	64520.41802871337	64520.26536165155
371	64874.55496	64856.27743217116	64875.48747991017	64874.9189953949	64874.957536454946	64874.89338972803
372	65230.02712	65211.7329882666	65231.01655699587	65230.45319121993	65230.47538235535	65229.67921008308
373	65586.82151	65568.16695349154	65587.52406842865	65586.965910569	65586.97159500873	65586.4592128526
374	65944.32969	65925.57935681296	65945.01004274654	65944.45718225642	65944.44620289402	65944.29735309897
375	66302.77943	66283.97022708155	66303.47450837286	66302.92703498085	66302.89923437574	66302.39038581392
376	66662.24602	66643.33959303249	66662.91749361705	66662.37549732604	66662.33071770471	66662.16562217975
377	67022.66748	67003.68748328624	67023.33902667536	67022.80259776163	67022.74068101884	67022.63520344146
378	67384.14198	67365.01392634926	67384.73913563164	67384.2083646439	67384.12915234384	67384.00104460168
379	67746.50821	67727.31895061485	67747.11784845813	67746.59282621644	67746.49615959403	67746.40481158509
380	68109.84148	68090.60258436379	68110.47519301607	68109.95601061103	68109.84173057301	68109.43539345406
381	68474.15573	68454.86485576515	68474.81119705655	68474.29794584827	68474.16589297442	68474.15725134646
382	68839.42687	68820.105792877	68840.1258882212	68839.61865983838	68839.46867438268	68839.47800089394
383	69205.74196	69186.32542364714	69206.41929404286	69205.91818038185	69205.75010227364	69205.7412345284
384	69572.98977	69553.52377591385	69573.69144194639	69573.1965351702	69573.01020401542	69573.00225151691
385	69941.21957	69921.70087740653	69941.94235924924	69941.45375178671	69941.24900686894	69941.30069760665
386	70310.42213	70290.8567557465	70311.1720731623	70310.68985770711	70310.46653798876	70310.53169685561
387	70680.60361	70660.99143844761	70681.38061079048	70680.90488030024	70680.6628244237	70680.66903159485
388	71051.77092	71032.10495291701	71052.56799913342	71052.09884682875	71051.83789311754	71051.93977115299
389	71423.91376	71404.1973264558	71424.73426508621	71424.27178444987	71423.9917709097	71424.11346578994
390	71797.03532	71777.26858625971	71797.87943544002	71797.42372021596	71797.12448453593	71796.72246989375

Continued on next page

Table S2 – *Continued from previous page*

n	E(n)	eqs. (6),(7)	eqs. (12),(13)	eqs. (14),(15)	eqs. (12),(17)	eq. (18)
391	72171.20986	72151.31875941982	72172.00353688278	72171.55468107527	72171.23606062893	72171.49028972157
392	72546.25833	72526.34787292313	72547.10659599984	72546.66469387259	72546.32652571904	72545.78327926857
393	72922.54111	72902.35595365336	72923.18863927465	72922.75378534987	72922.3959062349	72922.54263585768
394	73299.59334	73279.34302839148	73300.24969308937	73299.82198214692	73299.4442285041	73299.60105158256
395	73677.62848	73657.30912381645	73678.28978372552	73677.86931080207	73677.4715187538	73677.68215193746
396	74056.62463	74036.25426650584	74057.30893736468	74056.89579775275	74056.47780311135	74056.62382696246
397	74436.60046	74416.17848293643	74437.307180089	74436.90146933618	74436.46310760501	74436.61289463146
398	74817.5836	74797.08179948493	74818.28453788196	74817.88635178999	74817.42745816446	74817.6474292357
399	75199.53076	75178.96424242854	75200.24103662891	75199.85047125282	75199.37088062151	75199.54956134727
400	75582.44848	75561.82583794558	75583.17670211771	75582.79385376502	75582.29340071067	75582.11735913939
402	76351.1924	76330.48659092262	76351.98563598843	76351.61851161064	76351.0758362405	76350.81174866854
410	79465.74322	79444.30087442053	79466.39313263363	79466.09280636691	79465.37456460035	79465.9415682209
420	83446.99761	83424.7192534595	83447.55390292153	83447.3460010317	83446.39310006314	83447.04740417427
430	87526.11873	87503.10561416432	87526.68328305898	87526.57794691648	87525.37322698583	87526.33639569452
432	88353.70965	88330.54115088523	88354.26746743485	88354.18388190729	88352.92671422126	88353.73645455267
440	91703.32957	91679.48373071809	91703.80469032237	91703.81229279898	91702.33831422786	91703.40058861476
448	95115.54694	95091.15574264638	95116.0712976349	95116.17687044425	95114.47474595602	95115.85294357623
450	95978.44133	95953.87656183608	95978.94073799091	95979.07187627027	95977.3109283232	95978.77674055268
460	100351.7631	100326.30629634668	100352.11328032761	100352.37876907758	100350.3128783674	100351.76539752206
468	103920.8717	103894.85109965669	103921.25181738667	103921.6327150715	103919.30975791588	103921.2779972716
470	104822.8863	104796.79439528308	104823.343454114	104823.75431899584	104821.36525746541	104823.5045038923
480	109392.3187	109365.36163082179	109392.65171707066	109393.21918856264	109390.48848107201	109393.00655258191
482	110317.9966	110290.84635593255	110318.2844949619	110318.88465303989	110316.0833781093	110318.5791138175
490	114059.8426	114032.02812236542	114060.0578834578	114060.79339097234	114057.70232251787	114060.57919999765
492	115005.0933	114977.13505794645	115005.31256547487	115006.08306883185	115002.91766176392	115005.6718119888
500	118825.4625	118796.81337003359	118825.58115711618	118826.49632339155	118823.02594598169	118826.08466187029
510	123689.3168	123659.73628579623	123689.24016218046	123690.34679792965	123686.4779371394	123690.0746109854
520	128651.144	128620.81522245888	128651.0529716717	128652.36307047222	128648.076331697	128651.95715727133
522	129655.3225	129624.81130959367	129655.19554331925	129656.54778970512	129652.17512902805	129656.17516808667
530	133711.2618	133680.06800068647	133711.03713415348	133712.56286756325	133707.8386419911	133712.2029560135
540	138869.5943	138837.51193423342	138869.20969861723	138870.96341150315	138865.78188182248	138870.52814086748
550	144126.0928	144093.16385352978	144125.587237745	144127.5814438112	144121.9225896703	144127.1467964855
560	149480.8993	149447.04012775928	149480.1858696837	149482.43324718793	149476.27685042092	149482.03604201935
570	154933.7025	154899.1566855503	154933.0212784501	154935.53466609755	154928.86031573065	154934.99105464833
572	156036.2193	156001.3702029977	156035.37812419498	156037.94626835597	156031.1658613252	156037.41898837985
582	161607.1762	161571.3955351825	161606.1177850373	161608.96755204338	161601.64445048085	161608.51052658338
612	178909.7953	178871.17295822152	178908.0131549148	178911.78744881242	178902.71064417667	178911.25988716265
632	190936.2621	190895.91770703509	190934.14681067056	190938.60527914797	190928.25292667322	190938.00794049966
642	197097.1993	197055.79880301867	197094.71481193823	197099.53613000156	197088.513581785	197098.86940617018

Continued on next page

Table S2 – *Continued from previous page*

n	E(n)	eqs. (6),(7)	eqs. (12),(13)	eqs. (14),(15)	eqs. (12),(17)	eq. (18)
672	216169.994	216125.63155524989	216166.57569031793	216172.57018592168	216159.40546988556	216171.9976462898
732	256972.4358	256922.23282389558	256967.06971445505	256975.8025091358	256957.7480327573	256974.87268707206
752	271360.989	271308.6707933271	271354.75167062646	271364.51676905743	271344.6489109908	271363.6157599716
762	278703.061	278649.6069157894	278696.2989928849	278706.60314849275	278685.79361198336	278705.71612870845
792	301319.8734	301263.40324718703	301311.88387896935	301323.89806582936	301300.12206301215	301322.8762979923
812	316890.458	316831.8531479874	316881.4871111206	316894.7196965911	316868.84700175514	316893.67979105085
842	340985.6576	340923.5899661177	340974.8924871088	340990.0721477246	340960.8735608144	340988.9169143126
912	400657.6165	400587.79814550676	400642.6883648348	400662.9812973187	400625.1633431701	400661.725224001
932	418594.234	418522.1878407655	418578.0199557069	418599.92346014985	418559.41832745716	418598.5250144842
942	427710.4476	427637.3330076761	427693.62160764466	427716.35573823744	427674.4691213524	427714.89718352194
972	455651.0808	455574.65656985785	455632.25540047465	455657.5836839306	455611.39994533255	455655.7783709365
1152	641950.0016	641852.2311774394	641915.6805269591	641959.8921533293	641883.0012841929	641957.6278782754
1382	926576.1174	926448.1487237258	926513.433236739	926590.4684507988	926461.5600947391	926587.466077626
1500	1092900.864	1092756.820444641	1092820.3157621233	1092918.1606884976	1092756.781416969	1092914.2903774763
1632	1295282.238	1295119.829867032	1295178.9627536498	1295303.3880585537	1295100.9050135699	1295297.5401919608
1902	1762965.458	1762761.9941643886	1762803.9448113695	1762993.9298619984	1762691.2547429623	1762986.3414480253
2000	1950575.638	1950355.839684188	1950388.6719640114	1950606.2635002076	1950261.7578851255	1950599.644263972
2192	2345722.3	2345469.3449987066	2345479.6563318386	2345757.356651804	2345322.2956793746	2345750.031871169
2500	3055924.213	3055617.8577196025	3055578.6407148615	3055969.7742694453	3055365.2402655156	3055960.1071870513
2502	3060843.538	3060536.5747752627	3060496.980610021	3060888.9202949246	3060283.1870867247	3060879.6095508602
2832	3926826.088	3926458.3682294777	3926346.4106138824	3926883.8650261466	3926062.5643890672	3926871.864880887
3182	4963365.826	4962929.404603303	4962718.051155469	4963437.4296137905	4962348.541336872	4963423.048041085
3552	6191357.26	6190844.3591184635	6190501.620360463	6191444.848825719	6190028.711473323	6191426.918067982
3942	7632895.031	7632297.903998639	7631786.450687739	7633001.354446497	7631190.149151628	7632981.190899272
4352	9311276.278	9310584.709194215	9309861.489652226	9311402.175998578	9309119.410256281	9311378.14827127
**MSE**		**4300.80**	**11028.08**	**114.22**	**6186.41**	**73.23**
